# Borylation *via* iridium catalysed C–H activation: a new concise route to duocarmycin derivatives†

**DOI:** 10.1039/d4ob00814f

**Published:** 2024-06-18

**Authors:** Marco M. D. Cominetti, Zoë R. Goddard, Bethany R. Hood, Andrew M. Beekman, Maria A. O'Connell, Mark Searcey

**Affiliations:** a School of Pharmacy, University of East Anglia Norwich Research Park Norwich NR4 7TJ UK m.searcey@uea.ac.uk

## Abstract

The synthesis of the ethyl ester analogue of the ultrapotent antitumour antibiotic seco-duocarmycin SA has been achieved in eleven linear steps from commercially available starting materials. The DSA alkylation subunit can be made in ten linear steps from the same precursor. The route involves C–H activation at the equivalent of the C7 position on indole leading to a borylated intermediate 9 that is stable enough for peptide coupling reactions but can be easily converted to the free hydroxyl analogue.

## Abstract 2

A concise synthesis of an alkylation subunit of the duocarmycins is achieved *via* a C–H-activation approach. The ethyl ester analogue of duocarmycin SA can be made in eleven linear steps from a commercially available precursor.

## Graphical abstract

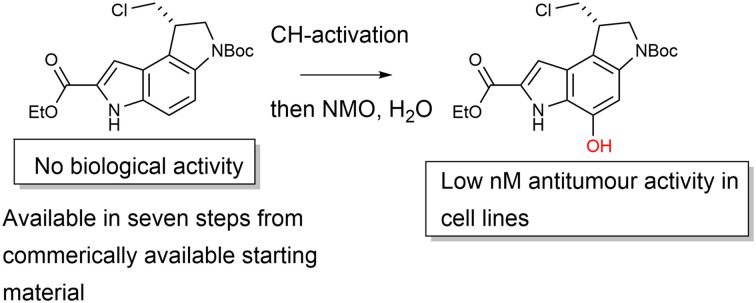

## Introduction

**Fig. 1:**
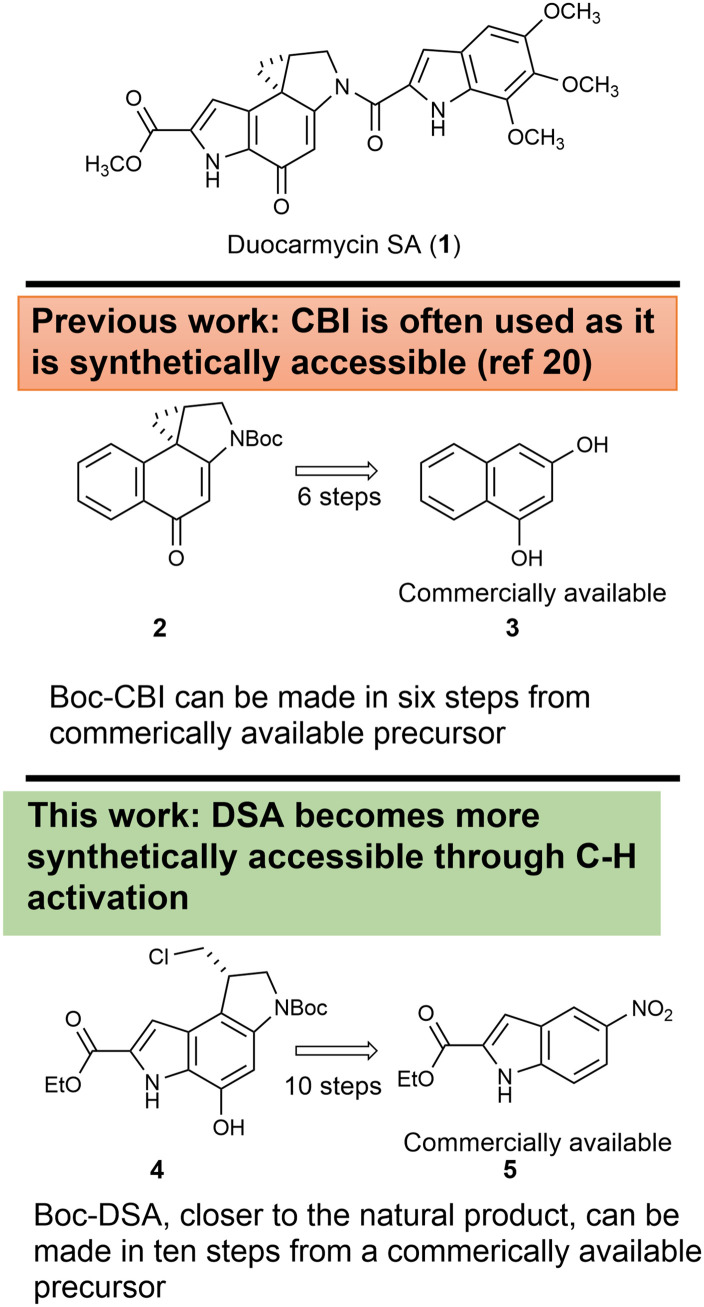
Duocarmycin SA (1). CBI (2) is often synthesised from 3. In this work, 4 is available from commercially available 5.

CC-1065,^1,2^ the duocarmycins^3^ such as duocarmycin SA (1, Fig. 1) and yatakemycin^4,5^ are amongst the most potent natural antitumour antibiotics that have been discovered. The densely substituted pyrroloindoline core of the natural products continues to interest synthetic chemists and there have been numerous reports of the total synthesis of the natural compounds and synthetic analogues. Efforts to translate this activity to clinical advantage have tended to focus on targeted systems through prodrugs with bioreductive,^6–8^ bio-oxidative,^9^ esterase^10,11^ or glycosylase enzymes^12–16^ or through antibody,^17^ peptide^18^ or small molecule conjugates.^19^ Many of these efforts have focussed on the use of the synthetic duocarmycin alkylation subunit known as CBI (2, CBI = 1,2,9,9*a*-tetrahydroCyclopropa[*c*]Benzo[*e*]Indol-4-one), as it is considered to be synthetically accessible, available in six steps from commercially available 1,3-dihydroxynaphthalene 3,^20^ whereas the routes to the natural products tend to be significantly longer. In this paper, we describe a new synthesis to the ethyl ester analogue 4 of the duocarmycin SA alkylating subunit in only ten linear steps from commercially available ethyl 5-nitroindole-2-carboxylate 5.

**Scheme 1:**
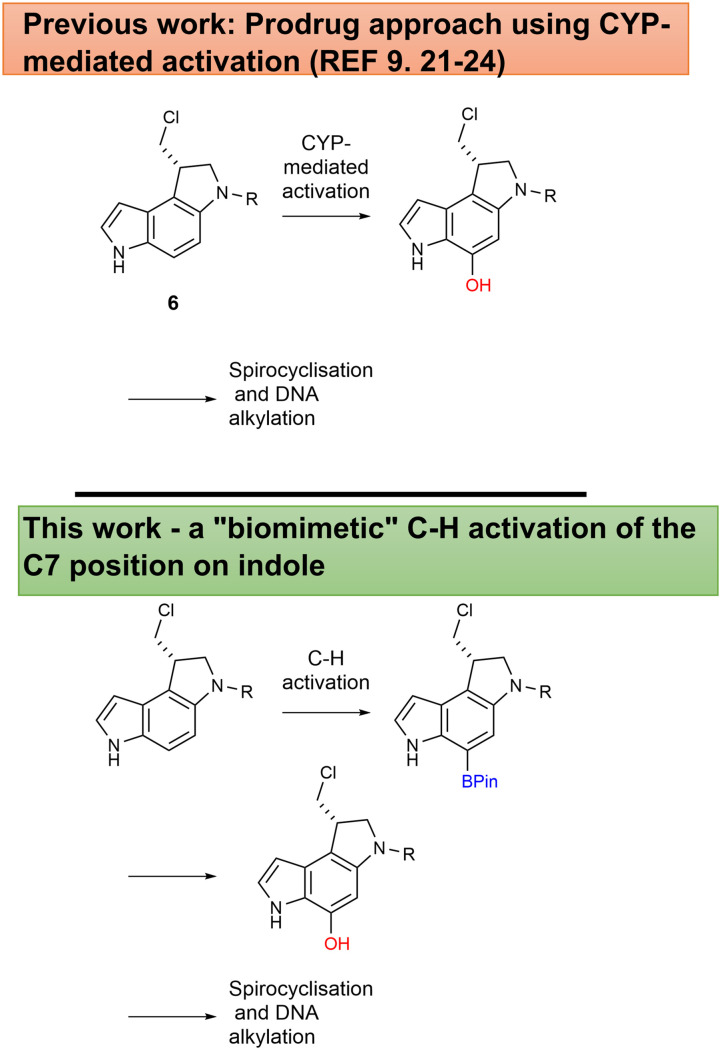
(A) CYP-enzyme-mediated prodrug activation.^5,11^ (B) Biomimietic approach using CH activation to generate the correct, active alkylating group structure.

In our efforts to generate bio-oxidatively activated prodrugs of the duocarmycins, we have previously shown that Cytochrome P450 (CYP) enzymes can oxidise de-hydroxylated chloromethylindoline derivatives such as 6 to their seco-CPI analogues (CPI = Cyclopropa[*c*]Pyrrolo[3,2-*e*]Indol-4(5*H*)one), which undergo spontaneous cyclisation and subsequent sequence selective DNA alkylation (Scheme 1).^9,21–24^ Direct hydroxylation *via non-enzymatic routes* to generate these derivatives is unreported, but we envisioned the possibility of introducing an intermediate boronic ester *via* C–H activation;^25^ this could be subsequently subjected to cross-coupling, or directly converted to the hydroxyl derivative at the final synthetic step (Scheme 1).^26^ Boronic acid derivatives are ideal coupling partners, suitable for Chan–Lam cross couplings, which allow convenient and mild C–O and C–N cross couplings,^27^ potentially opening possibilities for amino DSA derivatives (DSA = Duocarmycin SA alkylation subunit), but also direct conversion to hydroxyl groups. This transformation is mediated by different *N*-oxide derivatives and shows good conversions and functional group tolerance.^28^ Boronic esters have also been used within building blocks for peptide synthesis, demonstrating compatibility with the deprotections and amide couplings we intended to employ to make duocarmycin analogues.^29,30^

## Results

**Scheme 2:**
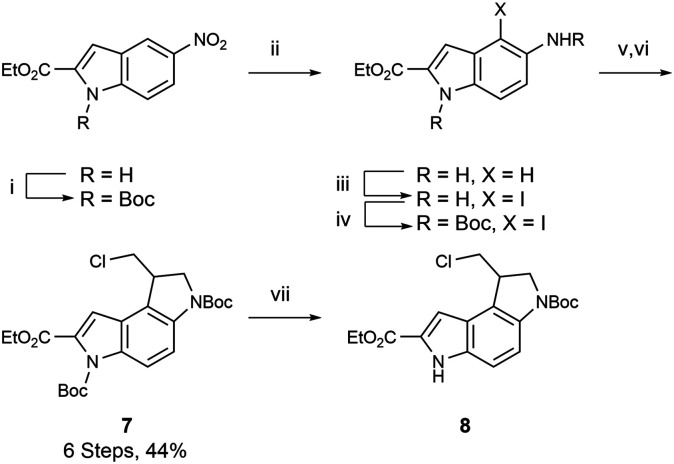
Synthesis of compound 8. (i) Boc_2_O, DMAP, CH_2_Cl_2_, RT; (ii) NH_4_Cl, Zn dust, THF, H_2_O, RT; (iii) NIS, DMF, 0 °C; (iv) Boc_2_O, Et_3_N, dioxane, reflux, 67% for 4 steps; (v) NaH (60% in mineral oil), anhydrous DMF, N_2_, 0 °C for 30 min, followed by 1,3-dichloropropene, RT, 88%; (vi) AIBN, TTMSS, degassed anhydrous toluene, N_2_, 90 °C, 74%; (vii) 5% piperazine in DMF, RT, 100% (NIS – *N*-iodosuccinimide, AIBN = azobisisobutyronitrile, TTMSS = tris(trimethylsilyl)silane).

Previous reports have highlighted the ability of [Ir(COD)(OMe)]_2_ complexes to directly borylate the 7-position on indoles *via* C–H activation under reasonably mild conditions.^31–33^ Of particular note, is the accessibility of the route, with both catalyst and ligand being readily available through common suppliers. As a starting point, we used compound 7 (Scheme 2), which we previously reported.^34^ It can be obtained in 44% yield over 6 steps (with 3 chromatographic purifications) on a multigram scale starting from commercially available ethyl 5-nitroindole-2-carboxylate.^34^ The methyl ester, mirroring the natural product, is not commercially available as far as we are aware, but it was unlikely that ultimately the ethyl ester group would have significant impact on the biological activity of analogues.

Due to the presence of the Boc group on the indole nitrogen and the suggested mechanism of C–H activation regioselectivity, which appears to be aided by coordination at the indole nitrogen,^31^ we decided to selectively deprotect this position. This can be readily achieved due to the unusual lability to nucleophilic bases of the Boc group on the indole nitrogen; the use of piperazine allows removal of the resulting Boc-protected piperazine by extraction, with quantitative recovery of product 8. Compound 8 was successfully resolved into two enantiomers using chiral flash chromatography (see ESI for methodology†).

**Scheme 3:**
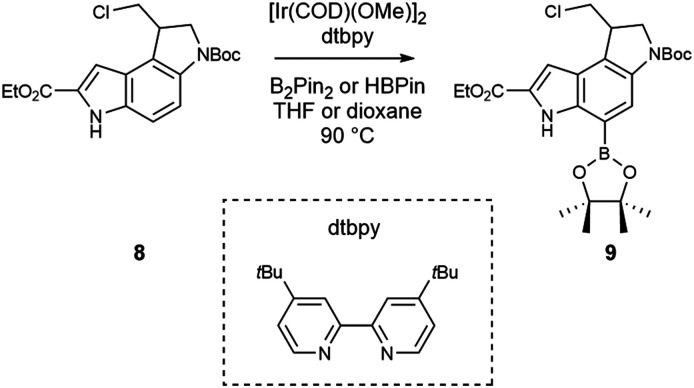
C7 borylation of compound 8.

The iridium catalysed borylation of indoles in the 7-position *via* C–H activation has been reported^32,33^ in either hexane, heptane, dioxane or THF, which led us to use THF to begin exploring optimal conditions, due to solubility limitations in the other solvents (Scheme 3). The reactions were carried out on 100–400 mg of starting material to evaluate isolated yields, and all starting material was extensively dried and stored in a desiccator. All reactions were set up in a glove box with anhydrous and degassed solvents in glass sealed vessels with PTFE lined septa; after preparation of the reaction mixture, the vials were transferred to a pre-heated block. We explored both pinacolborane and bis(pinacolato)diboron as donors, and both produced substantially identical results. In either case, and for all subsequent tests, we only observed product and unreacted starting material, without any noticeable side product. Interestingly, borane amount and catalyst loading did not affect the yield noticeably (Table 1). Doubling the reaction time also had limited effect on the yield, as did increasing the scale of the reaction. Dioxane was also tested as solvent, without any yield improvement. With only a variation of ∼10% in yield after these tests, it is apparent that different solvents, ligands and temperatures may have to be explored and further studies conducted to assess the cause of this limited conversion. Considering a 20–30% recovery of starting material that was then reusable, the reaction was then assessed on a preparative scale to complete the synthesis of the duocarmycin SA ethyl ester analogue.

**Table 1:** Screening of conditions for the borylation of 8. All reactions were in THF, except h which was performed in dioxane. In all cases the temperature was maintained at 90 °C. The dtbpy to [Ir(COD)(OMe)]_2_ ratio is 2 : 1. Yields are isolated yield after chromatographic separation

Entry	Cat. (equiv)	B_2_Pin_2_ (equiv.)	HBPin (equiv.)	Time (h)	Yield (%)	Scale (mg SM)
a	0.03	0.7		24	40	100
b	0.03		1.5	24	38	100
c	0.03	0.7		48	54	100
d	0.03	1.4		48	43	100
e	0.06	0.7		24	48	200
f	0.06	0.7		48	52	200
g	0.03	0.7		48	42	400
h	0.03	0.7		48	42	100

**Scheme 4:**
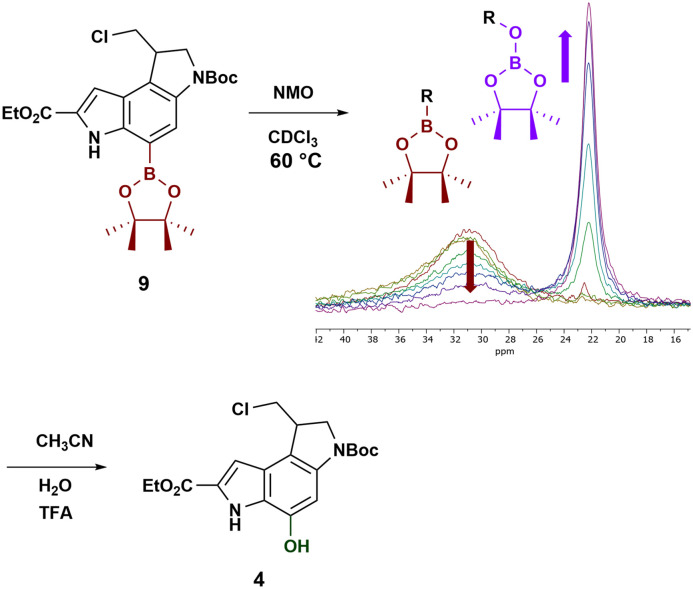
Monitoring the oxygen insertion into the C–B bond of 9 by ^11^B-NMR and subsequent conversion to compound 4 (90%) (NMO = *N*-methylmorpholine-*N*-oxide).

With compound 9 in hand, we explored the hydroxylation of the boronic ester. Zhu *et al.* reported a convenient open flask method using different *N*-oxides, and highlighted how boronic esters reacted more sluggishly as compared to boronic acids.^28^ In our case, the reaction did not proceed at an appreciable rate with the evaporation of the solvent, CH_2_Cl_2_, being a major issue. The use of less volatile chloroform allowed longer reaction times, but still without appreciable conversion. Performing the reaction in a sealed NMR tube allowed the use of a higher temperature (60 °C) while monitoring the reaction progress *via*^11^B-NMR (Scheme 4). In anhydrous conditions, the reaction halts after the oxygen insertion into the C–B bond (Scheme 4, centre), as described by Xie and colleagues.^35^ The resulting borate ester has a clearly distinct shift and is rapidly hydrolysed in the presence of water and acid, which also limits spurious cyclisation of the seco-derivative.

**Scheme 5:**
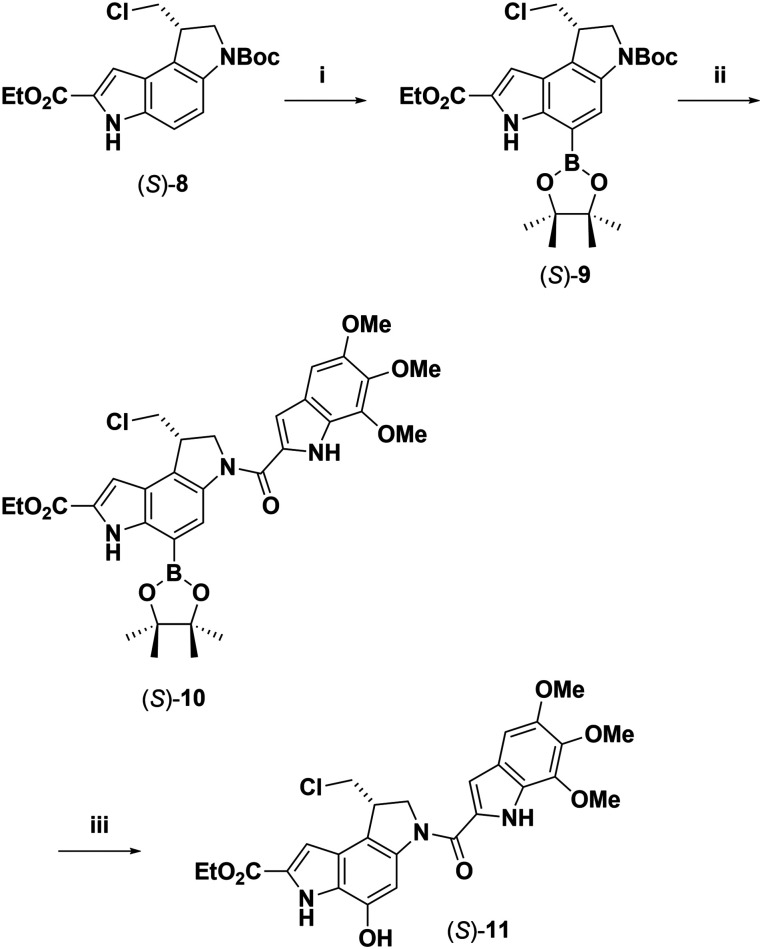
Synthesis of compound 11. (i) [Ir(COD)(OMe)], dtbpy, B_2_Pin_2_, THF, 90 °C, (9 52%, (*S*)-9 49%); (ii) TFA/CH_2_Cl_2_/TIPS, 45/45/10, RT; (ii) TMI-COOH, HATU, DIPEA, DMF, RT (10 48%, (*S*)-10 33%); (iii) NMO, CHCl_3_, 60 °C, RT then CH_3_CN, H_2_O, RT (12 55%, (*S*)-12 34%) (TFA = Trifluoroacetic acid, TIPS = Triisopropylsilane, HATU = Hexafluorophosphate Azabenzotriazole Tetramethyl Uronium, DIPEA = diisopropylethylamine).

After defining all required reactions, we proceeded to synthesise an analogue of duocarmycin SA. Following the previous C–H activation method, compound (*S*)-8 was converted to (*S*)-9 (Scheme 5) in 49% yield, which was subjected to Boc group deprotection and subsequent coupling with 5,6,7-trimethoxyindole-2-carboxylic acid (TMI-COOH) to give the product (*S*)-10 (33% yield compared with 48% for the racemic, no attempts were made to optimise yields). The main side product from this conversion was the hydrolysed boronic acid analogue (see 10A, ESI†). Hydroxylation of (*S*)-10 was effected as described for compound 9 to give the target ethyl ester analogue of seco-duocarmycin SA, compound (*S*)-11 (34% yield compared with 55% for racemic. No attempt was made to optimise yields).

The compounds were assessed for biological activity in HL-60 cells. Compounds 4, racemic 11 and (*S*)-11 demonstrated potent (nM to pM) activities in line with previously described derivatives (Table 2).^36^

**Table 2:** Cell proliferation assay, measured IC_50_

Compound	IC_50_ (nM) [95% CI]
9	763.6 [665.4–892.4]
4	26.88 [12.83–53.06]
Racemic 11	0.69 [0.61–0.79]
(*S*)-11	0.085 [0.078–0.094]

**Scheme 6:**
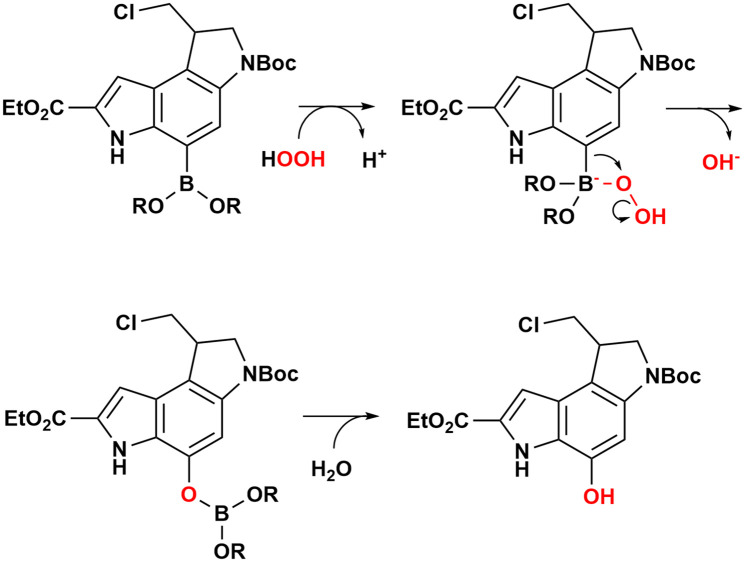
Putative mechanism for the oxidative deborylation mediated by hydrogen peroxide to form the active seco-derivative 9.

Interestingly, compound 9 showed more potent, sub-micromolar activity than was expected based upon its structure. Boron has an empty p orbital and therefore lacks the ability to spirocyclise, a requirement for potent biological activity in this family of compounds. Compound 9 is remarkably stable, both in open air and in solution, without displaying signs of oxidation to compound 4 (the sample used for biological testing was analysed by LC-MS to identify the potential presence of seco- and spiro-derivatives). On the other hand, boronic acids and esters are known to be subject to oxidation in a biological context,^37^ with reactive oxygen species mimicking the reaction with NMO, and leading to the formation of the seco-product, which in turn cyclises and alkylates DNA. Preliminary data seems to support this view and we are currently investigating methods to monitor this process in cells (Scheme 6).

## Conclusions

Trastuzumab duocarmazine, an antibody drug conjugate (ADC) that includes a duocarmycin as its payload, has progressed through Phase III clinical trials.^38^ It is apparent that the duocarmycin family of antitumour antibiotics have a role to play in the development of new therapeutics. Traditionally, analogues of the duocarmycins have focussed on CBI as the most accessible member of the family due to its synthetic tractability and similar potency to the natural products. The reported approach herein provides the shortest synthesis of a duocarmycin SA analogue to date and suggests that investigation of the natural product alkylation subunit as a central player in the design of new payloads for ADCs and for other targeted approaches is now accessible.

All the reagents used in this approach are commercially available, making the route immediately attractive. The key C–H alkylation in the 7-position of the indole analogue 8 is a late stage modification which has been applied for the first time to this family of derivatives, and provides a boronic ester which can be converted to the active seco-duocarmycin in a final oxidative step. We have also shown that simple chiral flash chromatography can be used to separate 8 into its enantiomers (see ESI†). The boronic ester is also a potential new pro-drug derivative and will be investigated further.

## Author contributions

MMDC: conceptualization, investigation, methodology, writing – original draft, writing – review and editing. ZRG: investigation, methodology, formal analysis, writing review and editing. BRH: investigation, formal analysis. AMB: conceptualization, methodology, writing – review and editing. MO'C: methodology, resources, writing – review and editing. MS: conceptualisation, funding acquisition, project administration, supervision, writing review and analysis.

## Data availability

The data supporting this article have been included as part of the ESI.†

## Conflicts of interest

There are no conflicts to declare.

## Supplementary Material

OB-022-D4OB00814F-s001
