# Supplementary material for: Borylation via iridium catalysed C–H activation: a new concise route to duocarmycin derivatives
Source: Org Biomol Chem. 2024 Jun 18;22(27):5603–7. doi: 10.1039/d4ob00814f (PMC11234497; doi:10.1039/d4ob00814f)
Supplement: OB-022-D4OB00814F-s001 [file OB-022-D4OB00814F-s001.pdf]

Electronic Supplementary Material (ESI) for Organic & Biomolecular Chemistry.
This journal is © The Royal Society of Chemistry 2024

Borylation via Iridium catalysed C-H activation: a new concise route to duocarmycin derivatives

Marco M. D. Cominetti, Zoë R. Goddard, Bethany R. Hood, Andrew M. Beekman, Maria A. O'Connell and Mark Searcey

School of Pharmacy, University of East Anglia, Norwich Research Park, Norwich NR4 7TJ, UK.

m.searcey@uea.ac.uk

Contents

Index of Figures 1
General methods 3
Compound **8** 3
Isolation of (*S*)-**8** 7
Compounds **9** and (*S*)-**9** 9
Compounds **10**, **10A** and (*S*)-**10** 14
Data for **10** and (*S*)-**10** 14
Data for **10A** 20
Compound **4** 25
Compound **11** and (*S*)-**11** 30
Proliferation assay 35
References 36

Index of Figures

Figure 1. ^{1}H-NMR of **8** and zoom in on regions of interest. 4
Figure 2. ^{13}C-NMR of **8**. 4
Figure 3. HSQC of compound **8**. H-C correlation for C11, missing from the ^{13}C spectra, has been picked for clarity. 5
Figure 4. HMBC of compound **8**. H-C correlations for C5, C6 and C20, missing from the ^{13}C spectra, have been picked for clarity. 5
Figure 5. COSY of compound **8**. 6
Figure 6. NOESY of compound **8**. Correlation between H7-H12 which was used to identify H12 is picked for clarity. 6
Figure 7. HRMS of compound **8**. 7
Figure 8. Representative trace of racemic **8**. Red is 214 nm, blue 254 nm, and black is λ-all (200-400 nm). Black line with percentage represents the concentration of IPA. Numbered slices correspond to individual fractions. 7
Figure 9. Trace of (*S*)-**8**, collected from fraction 3 to 5 of sample in Figure 8. Red is 214 nm, blue 254 nm, and black is λ-all (200-400 nm). Black line with percentage represents the concentration of IPA. 8
Figure 10. Trace of (*R*)-**8**, collected from fraction 7 to 9 of sample in Figure 8. Red is 214 nm, blue 254 nm, and black is λ-all (200-400 nm). Black line with percentage represents the concentration of IPA. 8
Figure 11. ^{1}H-NMR of **9** and zoom in on regions of interest. 10
Figure 12. ^{13}C-NMR of **9**. 10
Figure 13. HSQC of compound **9**. 11

Figure 14. HMBC of compound **9**....11
Figure 15. COSY of compound **9**. 12
Figure 16. NOESY of compound **9**....12
Figure 17. ^{11}B-NMR of compound **9**....13
Figure 18. HRMS of compound **9**. 13
Figure 19. ^{1}H-NMR of **10** and zoom in on regions of interest....16
Figure 20. ^{13}C-NMR of **10** and zoom in on regions of interest. 16
Figure 21. HSQC of compound **10**....17
Figure 22. HMBC of compound **10**....17
Figure 23. COSY of compound **10**. 18
Figure 24. NOESY of compound **10**....18
Figure 25. 11B-NMR of compound **10**. 19
Figure 26. HRMS of **10**. 19
Figure 27. ^{1}H-NMR of **10A** and zoom in on regions of interest. 23
Figure 28. ^{13}C-APT of **10A**. 23
Figure 29. HSQC of compound **10A**. 24
Figure 30. HMBC of compound **10A**. 24
Figure 31. COSY of compound **10A**....25
Figure 32. NOESY of compound **10A**. 25
Figure 33. 11B-NMR of compound **10A**....26
Figure 34. HRMS of **10A**....26
Figure 35. ^{1}H-NMR of **4** and zoom in on regions of interest....28
Figure 36. ^{13}C-NMR of **4**....28
Figure 37. HSQC of compound **4**....29
Figure 38. HMBC of compound **4**....29
Figure 39. COSY of compound **4**. 30
Figure 40. NOESY of compound **4**....30
Figure 41. HRMS of compound **4**. 31
Figure 42. ^{1}H-NMR of **11** and zoom in on regions of interest....33
Figure 43. ^{13}C-NMR of **11**. 33
Figure 44. HSQC of compound **11**....34
Figure 45. HMBC of compound **11**....34
Figure 46. COSY of compound **11**. 35
Figure 47. NOESY of compound **11**....35
Figure 48. HRMS of compound **11**....36

General methods

All chemicals were reagent or analytical grade and were purchased from Merck, Fluorochem and Fisher Scientific. NMR data was recorded on Bruker spectrometers using the specified deuterated solvent. Data processing was performed using Bruker TopSpin 3.5 and MestReNova 14. The chemical shifts for both ^{1}H- and ^{13}C- were recorded in ppm and were referenced to the residual solvent peak. Accurate mass spectra were recorded using the services provided by the Science Analytical Facility, UEA. Flash chromatography was performed on either an Advion interchim puriFlash, Teledyne ISCO or Biotage Isolera Four system, with either MODUS or puriFlash columns, as specified for each individual compound.

Compound 8

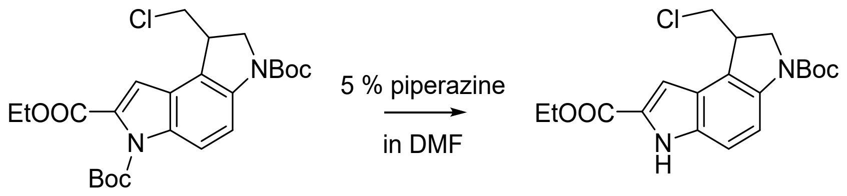

Compound **7** (7.71 g, 16.1 mmol) was dissolved in 200 mL of 5 % piperazine in DMF and stirred at room temperature for 5 hours. The solution was then diluted with 1 L of 1 M HCl and extracted five times with ethyl acetate. The organic layers were collected, washed with water, brine, and then dried over sodium sulfate. The solvent was evaporated under reduced pressure to give **8** as a white to yellow solid (6.1 g).

Yield = 100 %

HRMS: $[M+H]^+$ m/z = 379.1325 (calculated = 379.1425)

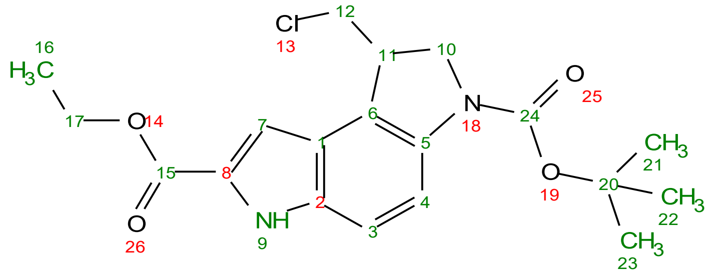

^{1}H NMR (400 MHz, DMSO-d_6) δ 11.91 (d, J = 2.2 Hz, 1H, 9), 7.87 (s, 1H, 4), 7.34 (d, J = 8.8 Hz, 1H, 3), 7.24 (dd, J = 2.2, 0.9 Hz, 1H, 7), 4.39 – 4.30 (m, 2H, 17), 4.19 – 4.04 (m, 3H, 10', 12', 11), 4.02 – 3.94 (m, 1H, 12''), 3.90 (dd, J = 10.7, 4.1 Hz, 1H, 11''), 1.51 (s, 9H, 21, 22, 23), 1.34 (t, J = 7.1 Hz, 3H, 16).

^{13}C NMR (101 MHz, DMSO) δ 161.18 (15), 151.61 (24), 136.66 (HMBC, 5), 134.66 (2 or 8), 128.43 (2 or 8), 123.34 (1), 120.94 (HMBC, 6), 113.25 (4), 112.18 (3), 104.93 (7), 79.65 (HMBC, 20), 60.47 (17), 51.71 (10), 47.44 (12), 41.12 (HSQC, 11), 28.10 (21, 22, 23), 14.30 (16).

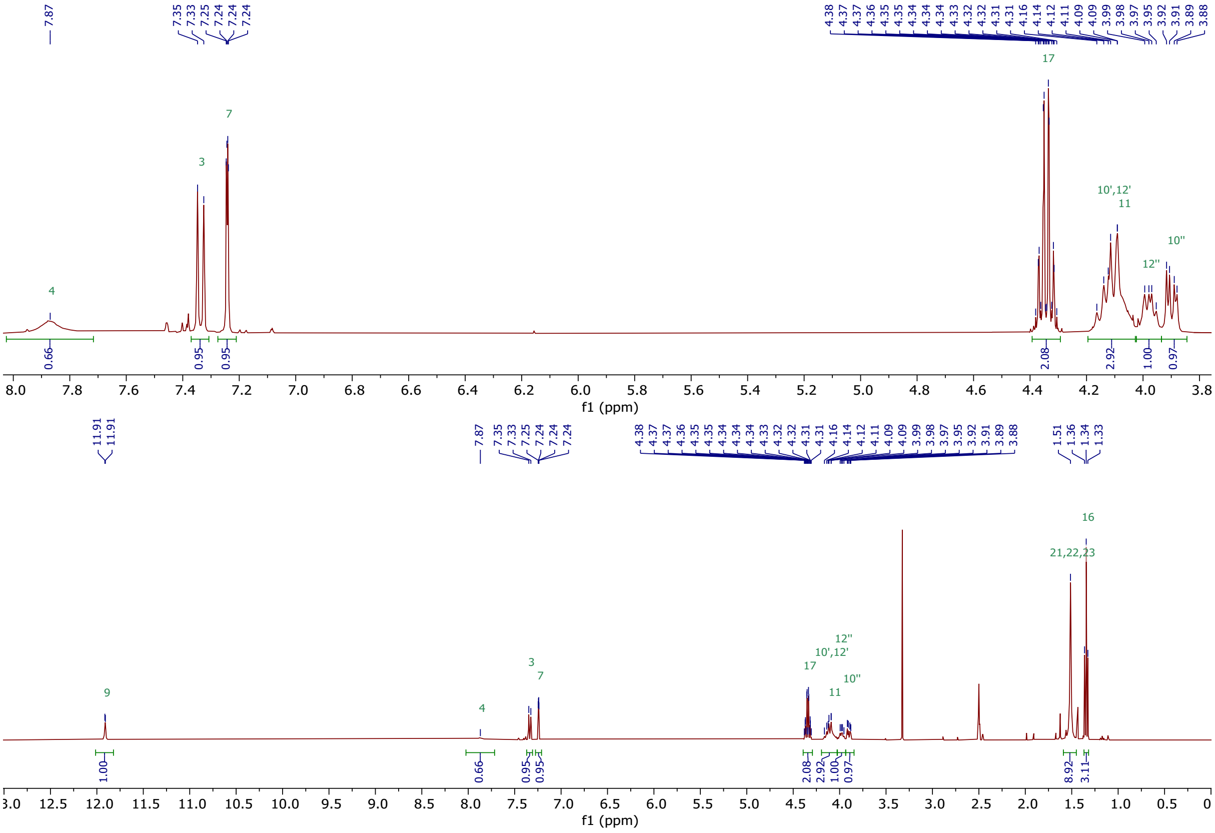

Figure 1. ¹H-NMR of 8 and zoom in on regions of interest.

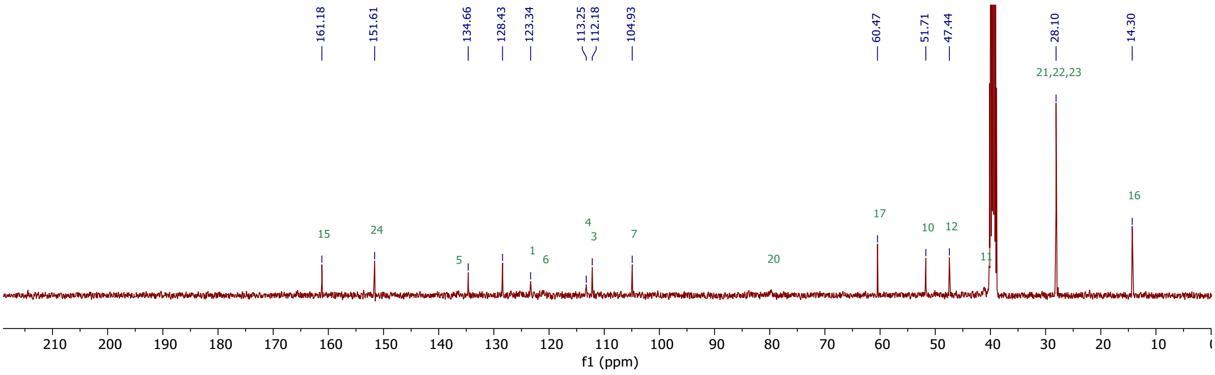

*Figure 2. ¹³C-NMR of **8**.*

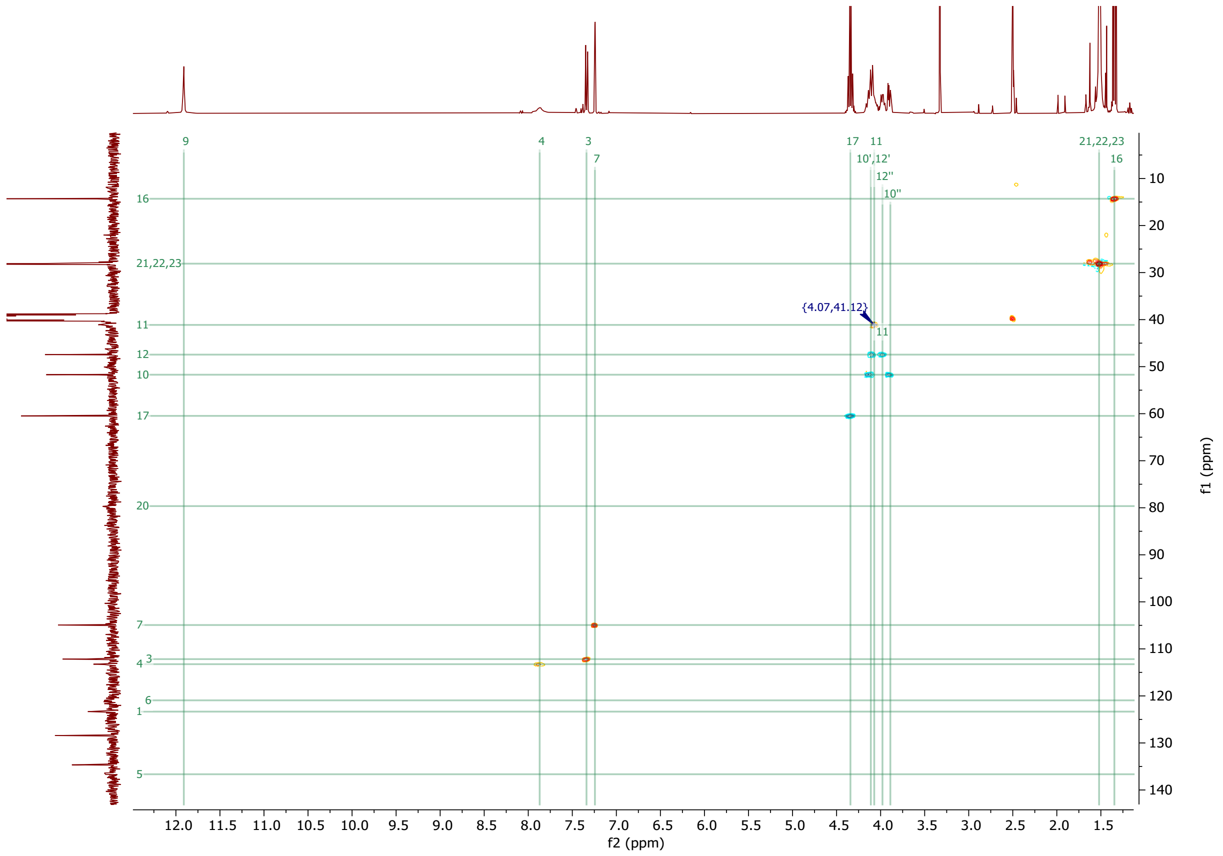

*Figure 3. HSQC of compound **8**. H-C correlation for C11, missing from the ^{13}C spectra, has been picked for clarity.*

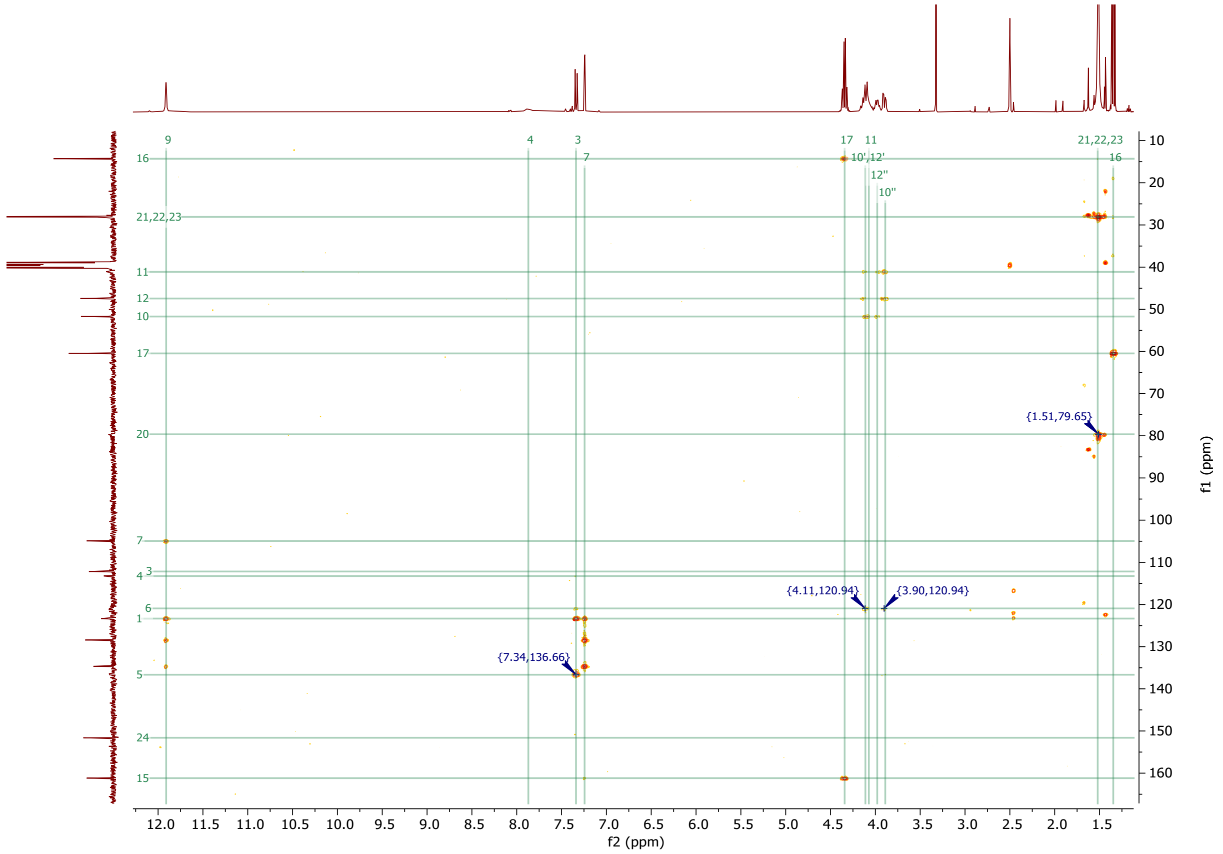

*Figure 4. HMBC of compound **8**. H-C correlations for C5, C6 and C20, missing from the ^{13}C spectra, have been picked for clarity.*

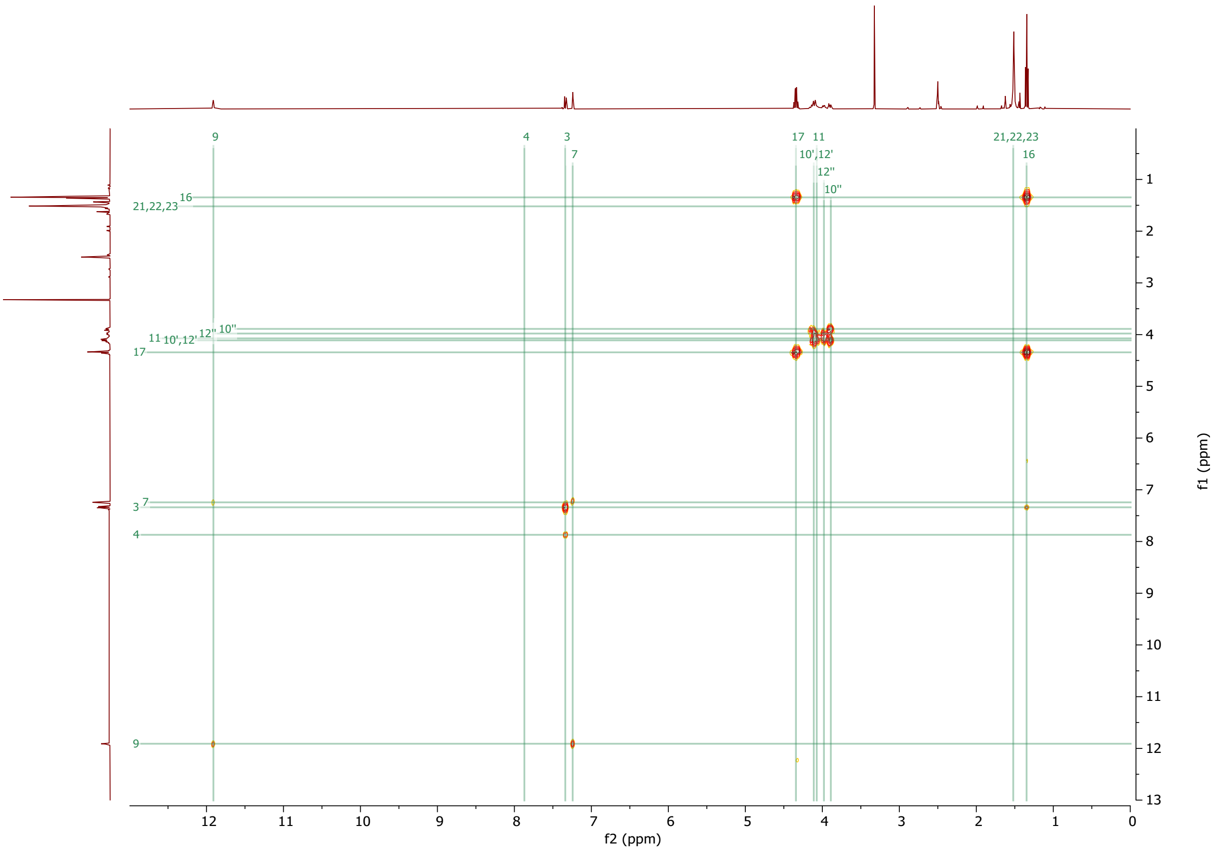

*Figure 5. COSY of compound **8**.*

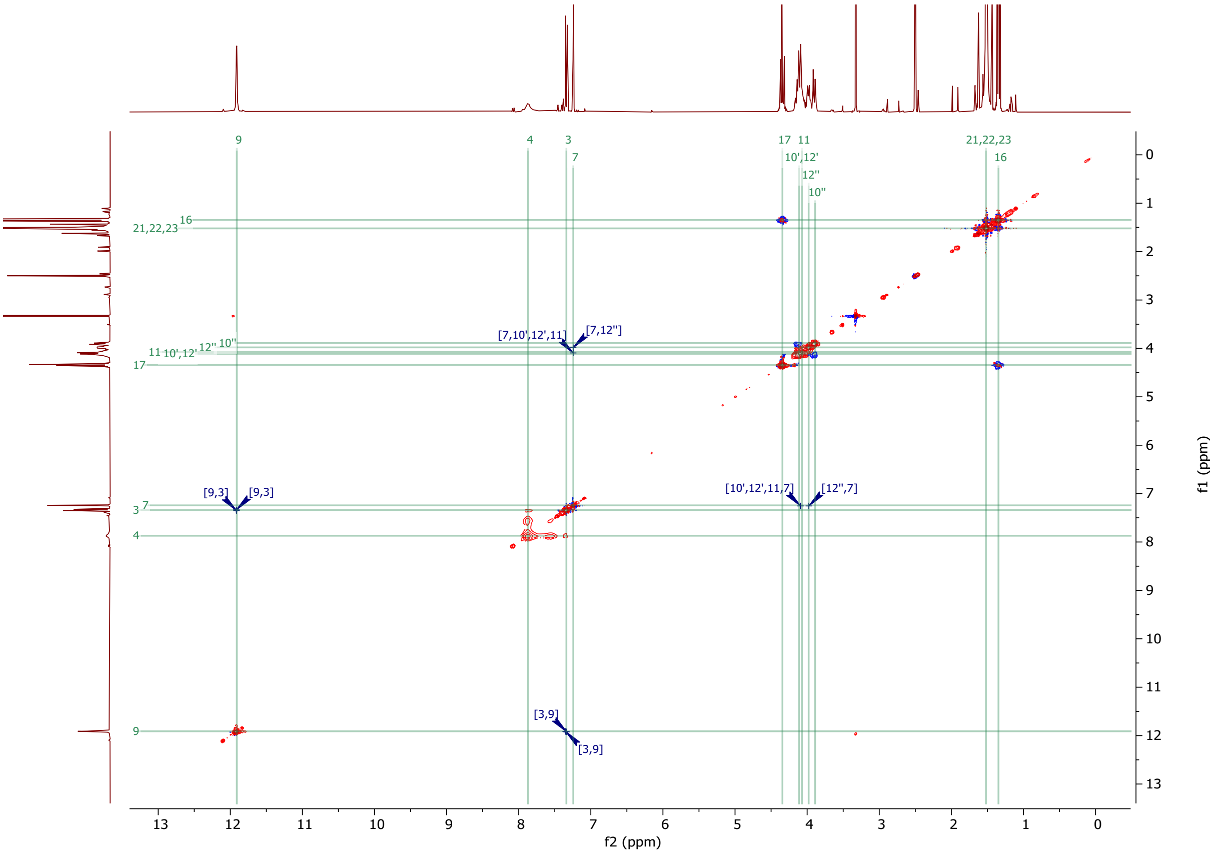

*Figure 6. NOESY of compound **8**. Correlation between H7-H12 which was used to identify H12 is picked for clarity.*

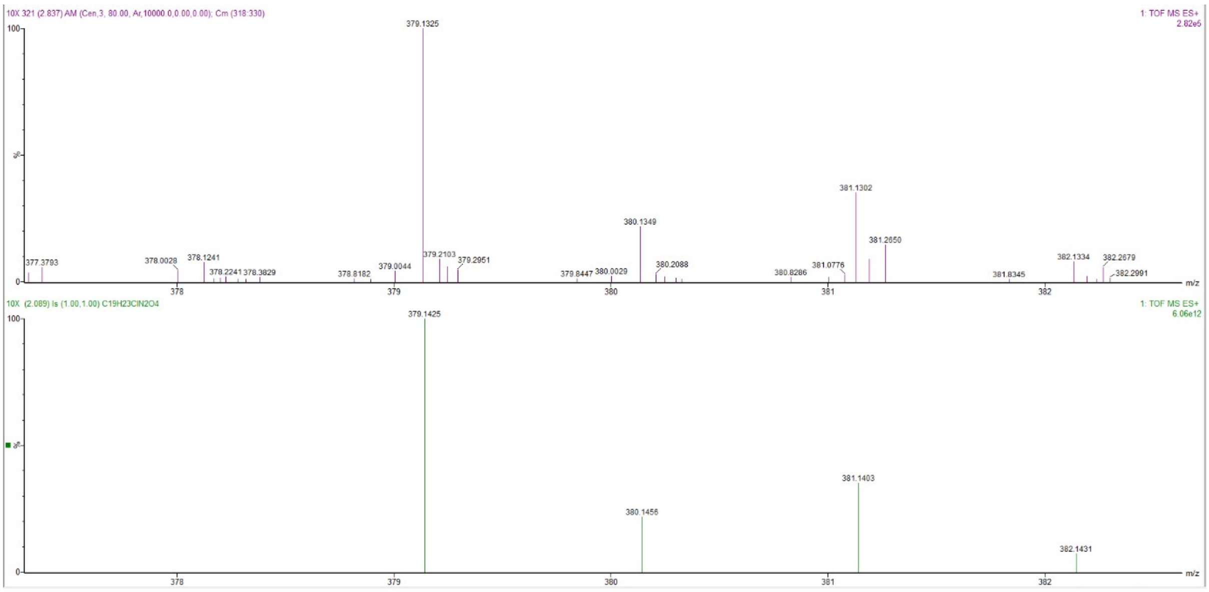

*Figure 7. HRMS of compound **8**.*

Isolation of (*S*)-8

Compounds **8** was dry loaded (90-100 mg dissolved in DCM and dried with 400 mg of silica) on a 25 g Puriflash Chiral IA column and eluted with 10 % IPA in Hexane on a Biotage Selekt. The first peak eluted is (*S*)-**8**. On this scale, due to solubility issues, only the S enantiomer can be isolated in reasonable purity and yield. Purity is assessed as described below.

The racemic mixture and the products were analysed by reinjecting ~5 mg of product, dissolved in warm IPA, on a 4 g Puriflash Chiral IA column and eluted with 10 % IPA in Hexane on a Biotage Selekt. Under these conditions, both enantiomers can be isolated and analysed (Figure 8, Figure 9, Figure 10).

(S)-**8** isolated yield of about 27 % when working on 90-100 mg batches

(S)-**8** α_D^{21} -35.25 (c = 1.986 mg/mL, $CHCl_3$)

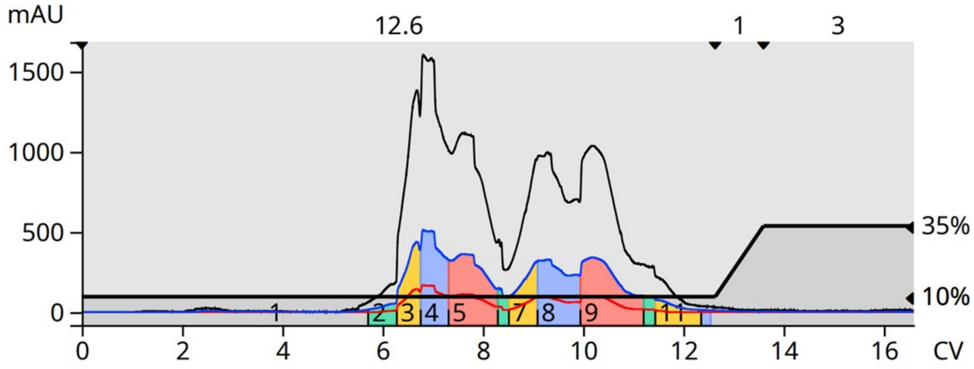

*Figure 8. Representative trace of racemic **8**. Red is 214 nm, blue 254 nm, and black is λ-all (200-400 nm). Black line with percentage represents the concentration of IPA. Numbered slices correspond to individual fractions.*

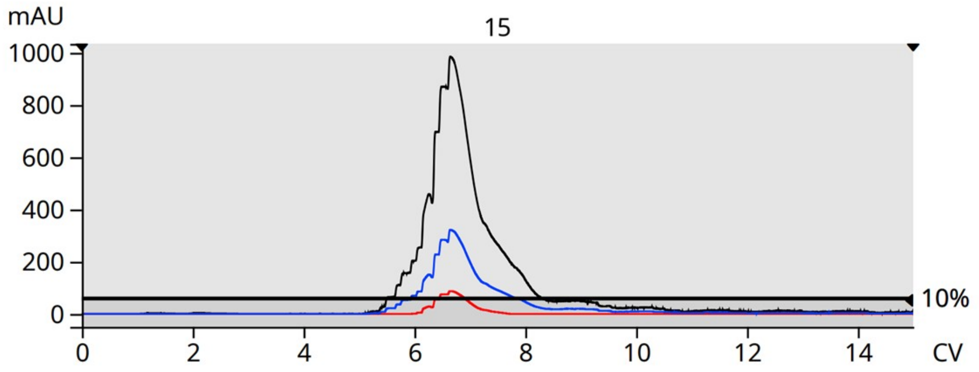

*Figure 9. Trace of (S)-**8**, collected from fraction 3 to 5 of sample in Figure 8. Red is 214 nm, blue 254 nm, and black is λ-all (200-400 nm). Black line with percentage represents the concentration of IPA.*

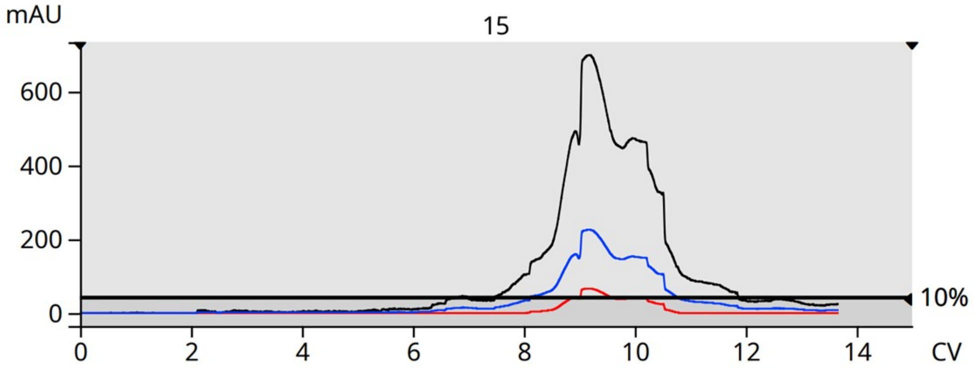

*Figure 10. Trace of (R)-**8**, collected from fraction 7 to 9 of sample in Figure 8. Red is 214 nm, blue 254 nm, and black is λ-all (200-400 nm). Black line with percentage represents the concentration of IPA.*

Compounds 9 and (*S*)-9

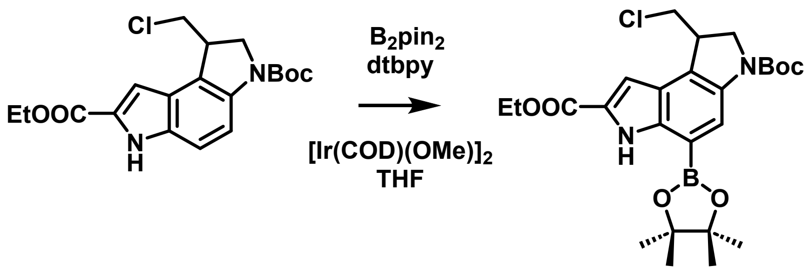

The following method is an example for this procedure. All other reactions to synthesise 9 and (S)-9 follow this procedure with the parameters specified in Table 1 of the paper and in the text.

In a glove box, dtbpy (8.5 mg, 0.032 mmol) was dissolved in 1 mL of anhydrous, degassed THF. $[Ir(COD)(OMe)]_2$ (10.5 mg, 0.016 mmol) was dissolved in the previous solution and then mixed with B_2Pin_2 (93.8 mg, 0.37 mmol). The solution, which slowly turns from yellow to red, was added to **8** (200 mg, 0.53 mmol). The tubes with the previous materials were washed with 1 mL of anhydrous, degassed THF, which was then added to the reaction. The reaction vial was sealed with a PTFE-lined septum, removed from the glove box, and stirred on a pre-heated heating block (temperature measured at the block = 90 °C) for 48 hours. The solution was cooled down, the solvent was evaporated and the residue was dissolved in a minimum amount of DCM. The solution was directly injected on a MODUS eTwist 25g column and purified with a linear gradient from 0 to 25 % of ethyl acetate in hexane over 15 column volumes, at a flow rate of 25 mL/min. The product was obtained as a white to light yellow solid (138.1 mg).

9 α_D^{21} 0 (c = 3.63 mg/mL, $CHCl_3$)

(*S*)-**9** (synthesised from (S)-8) α_D^{21} -27.5 (c = 2.181 mg/mL, $CHCl_3$)

Yield **9** = 52 %

Yield (*S*)-**9** = 49 %

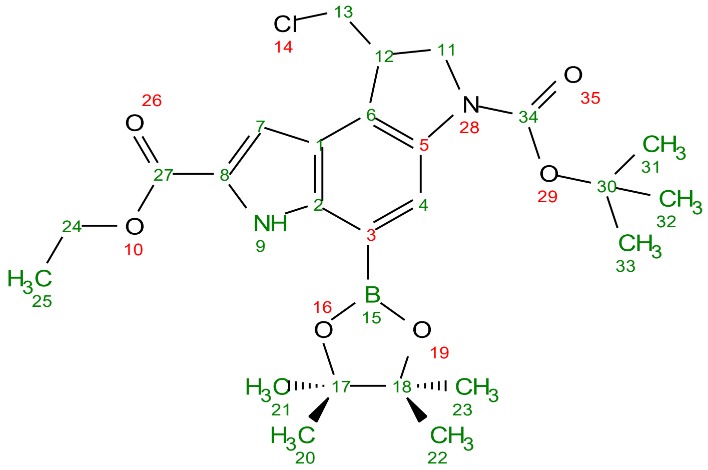

^{1}H NMR (400 MHz, Acetone-d6) δ 9.76 (s, 1H, 9), 8.50 (s, 1H, 4), 7.29 (d, J = 2.1 Hz, 1H, 7), 4.39 (qd, J = 7.1, 0.7 Hz, 2H, 24), 4.26 – 4.17 (m, 2H, 11’, 12), 4.17 – 4.13 (m, 1H, 13’), 4.12 – 4.04 (m, 1H, 11’’), 4.00 – 3.91 (m, 1H, 13’’), 1.57 (s, 9H, 31, 32, 33), 1.44 (s, 12H, 20, 21, 22, 23), 1.39 (t, J = 7.1 Hz, 3H, 25).

^{13}C NMR (101 MHz, Acetone) δ 162.00 (27), 152.91 (34), 140.05 (2), 129.60 (8), 126.66 (6), 124.27 (1), 121.49 (4), 105.55 (7), 85.10 (17, 18), 61.59 (24), 52.73 (11), 47.79 (13), 42.98 (12), 28.61 (31, 32, 33), 25.25 (20, 21, 22, 23), 14.63 (25).

^{11}B NMR (128 MHz, Acetone) δ 31.05.

HRMS: $[M+H]^+$ m/z = 505.2277 (calculated = 505.2281)

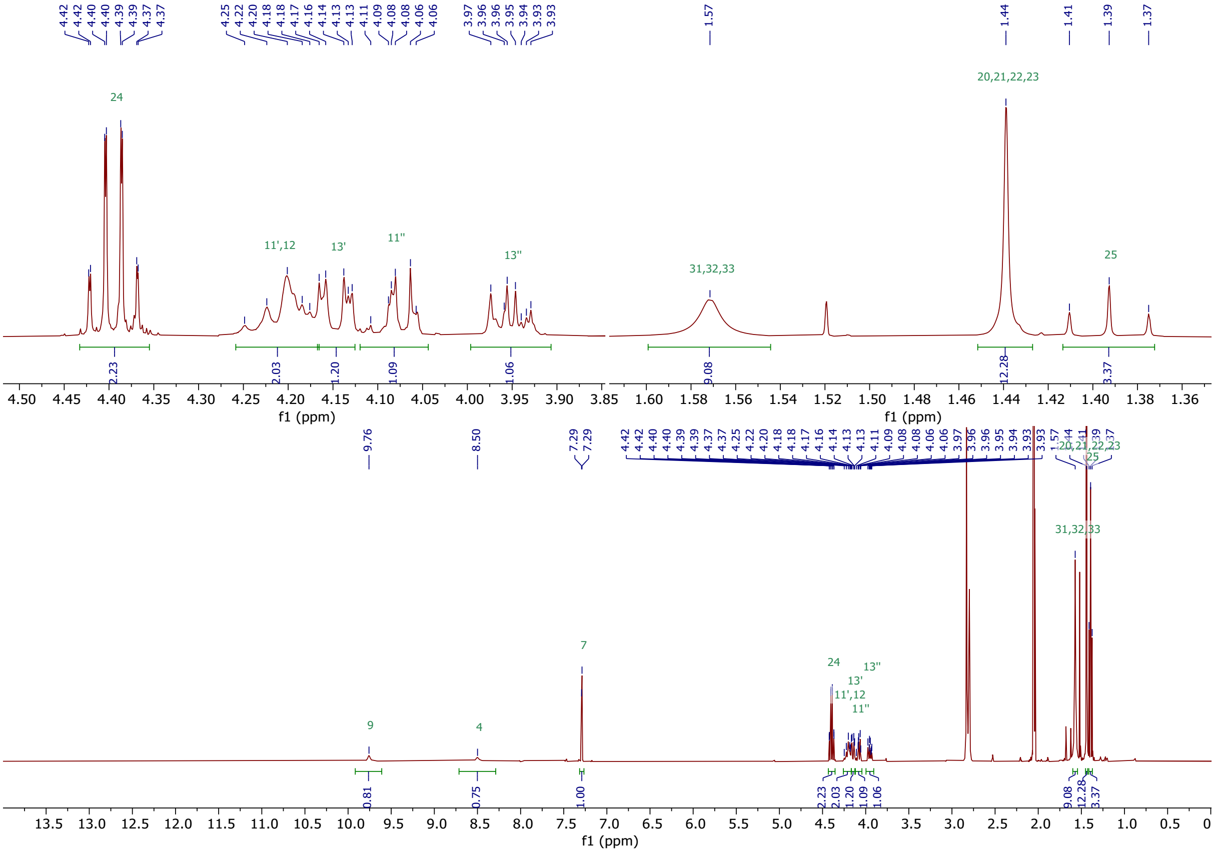

*Figure 11. ^{1}H-NMR of **9** and zoom in on regions of interest.*

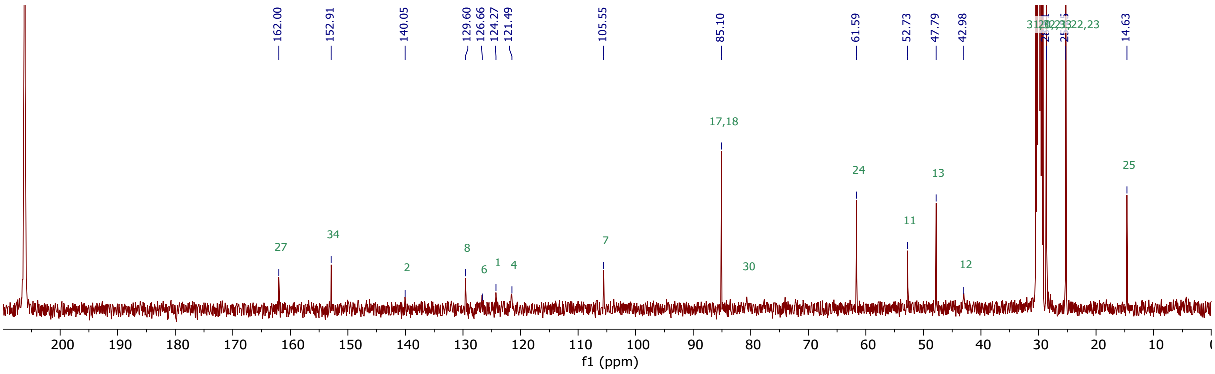

*Figure 12. ^{13}C-NMR of **9**.*

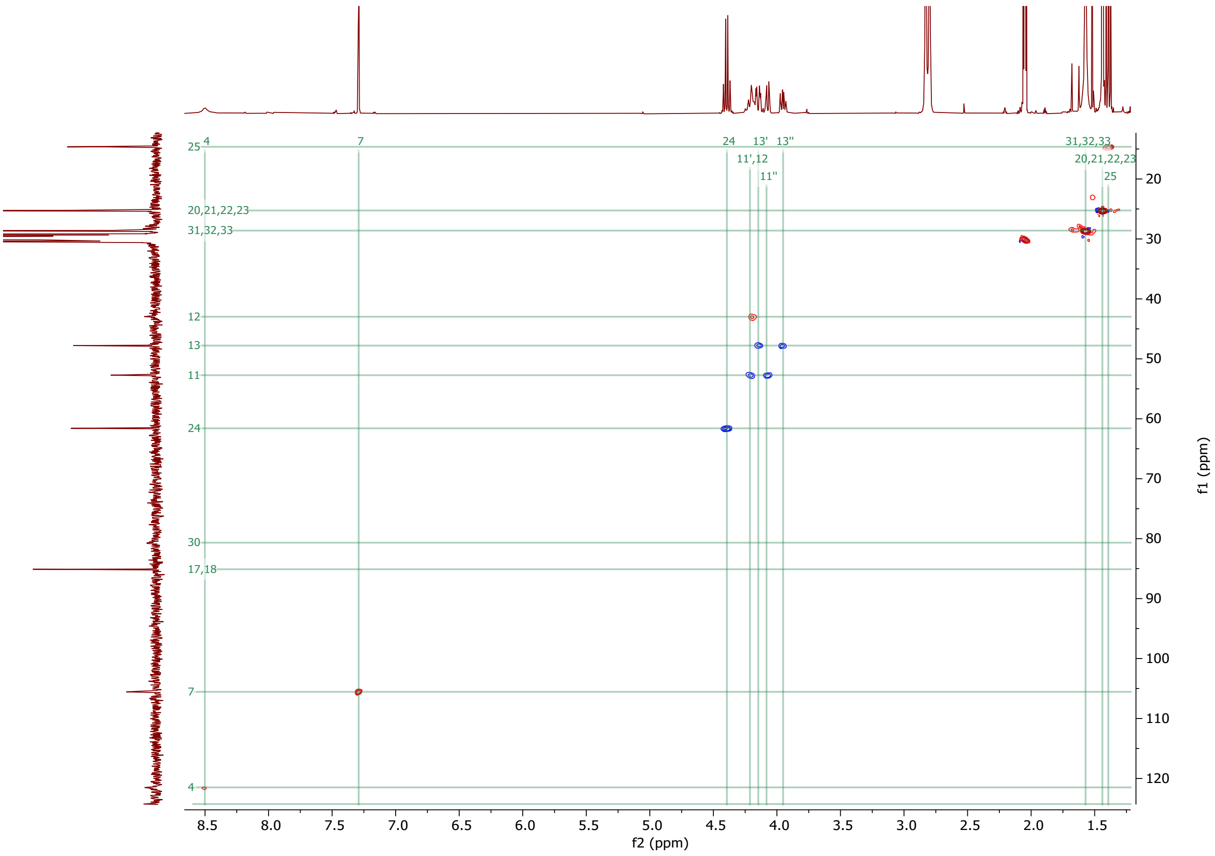

*Figure 13. HSQC of compound **9**.*

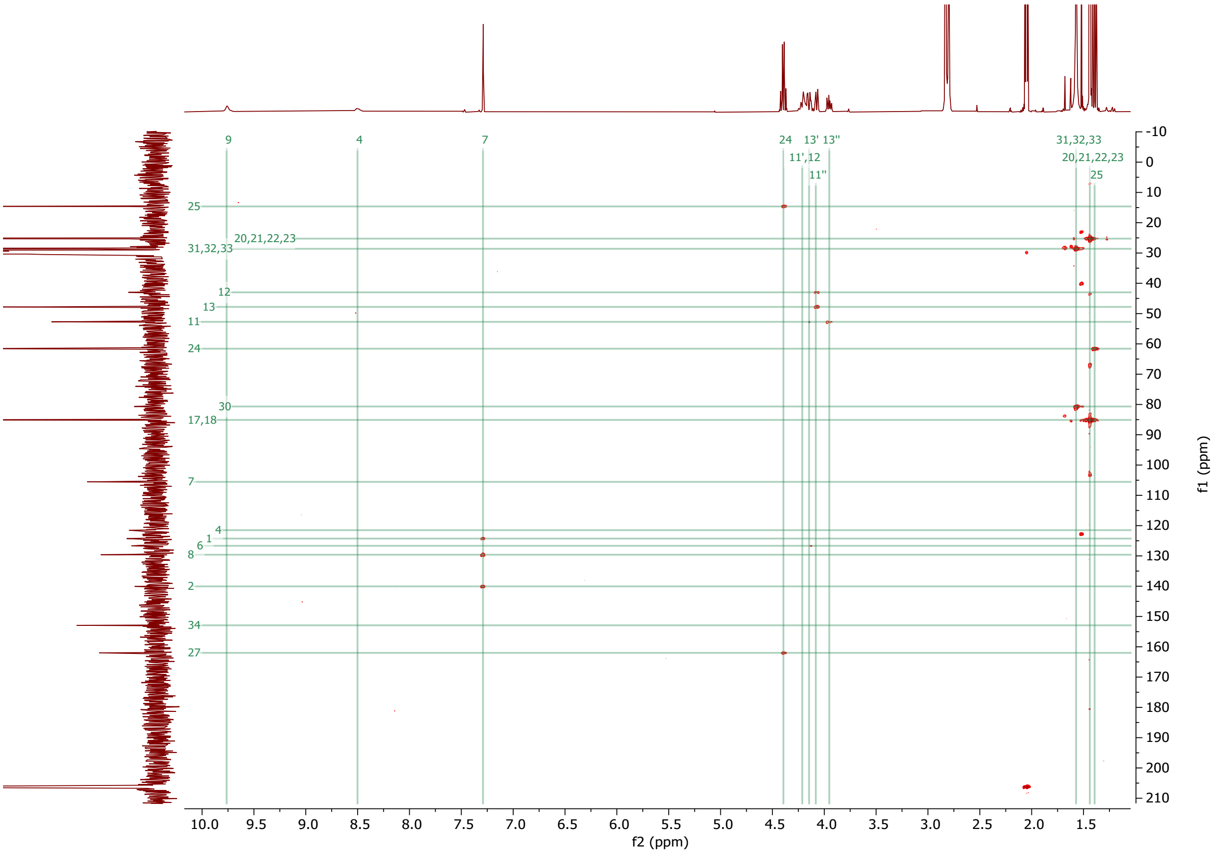

*Figure 14. HMBC of compound **9**.*

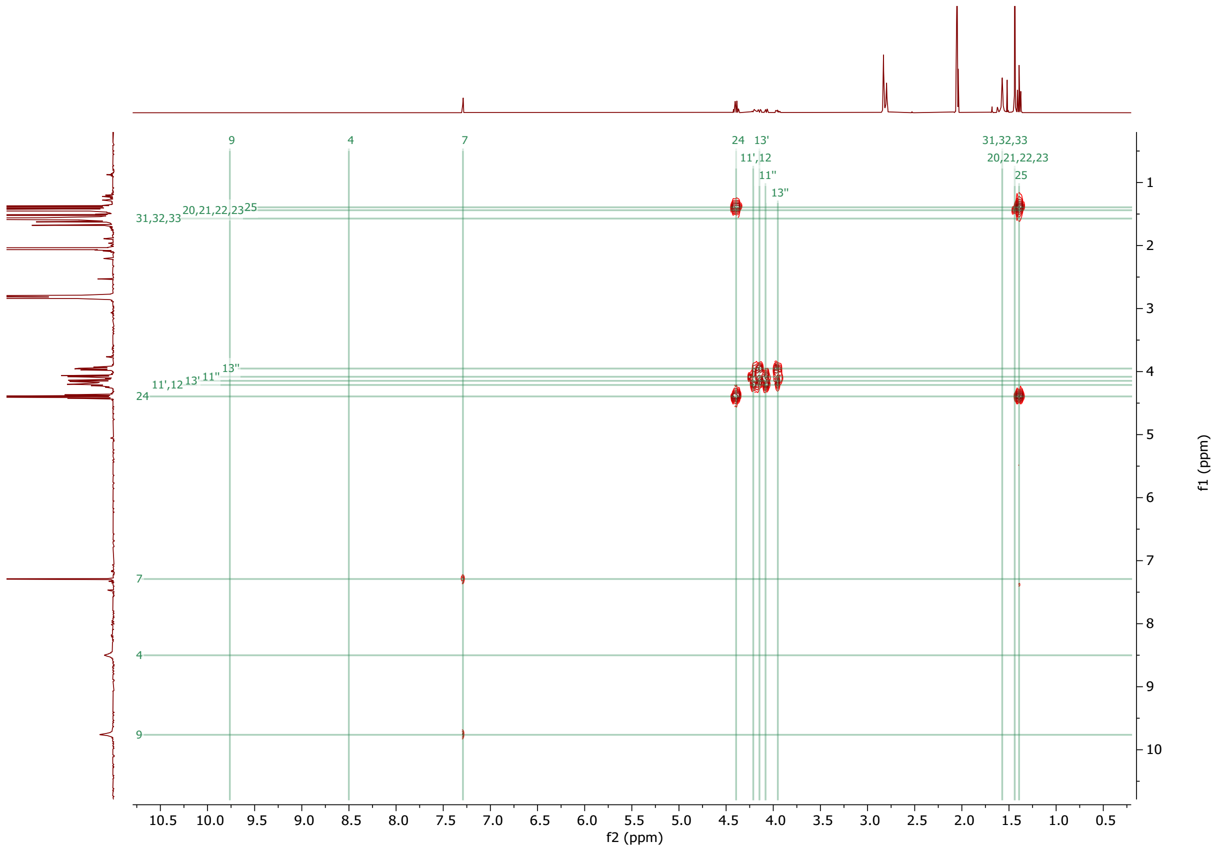

*Figure 15. COSY of compound **9**.*

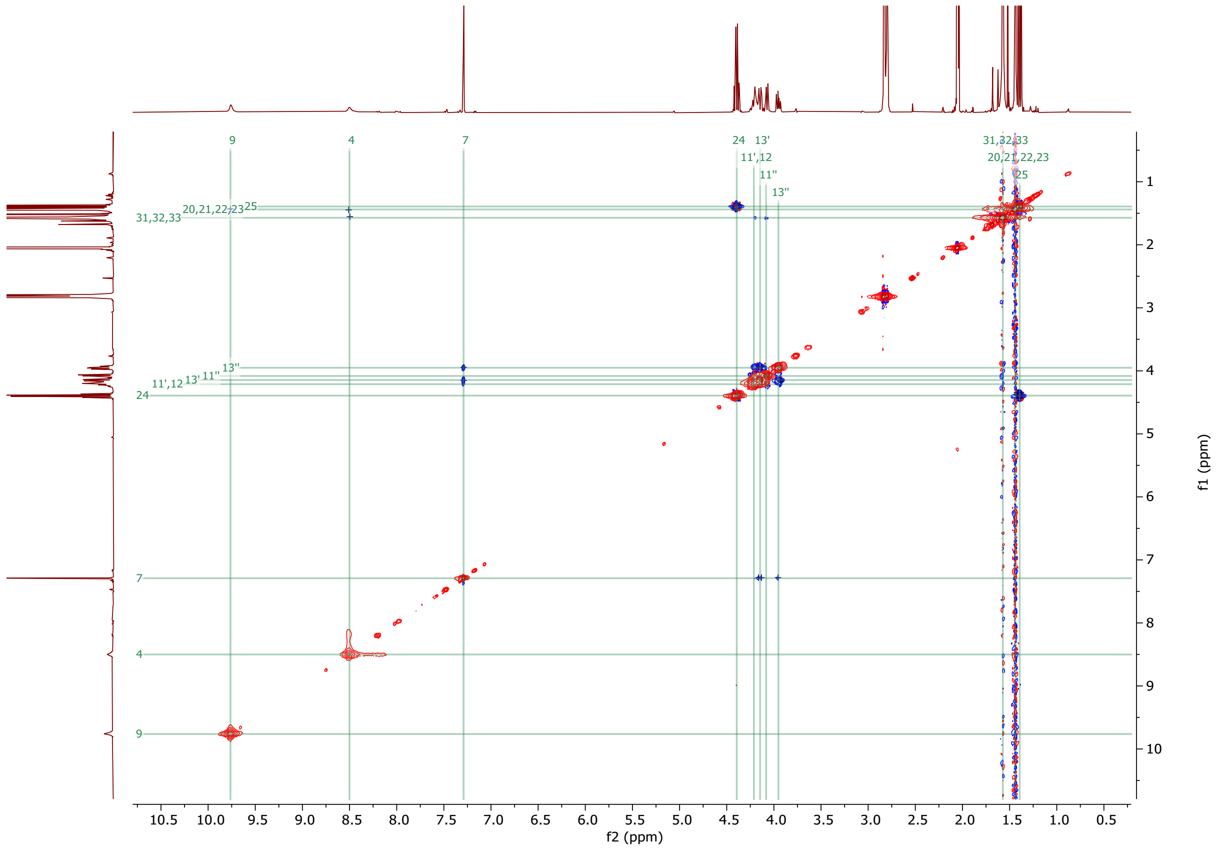

*Figure 16. NOESY of compound **9**.*

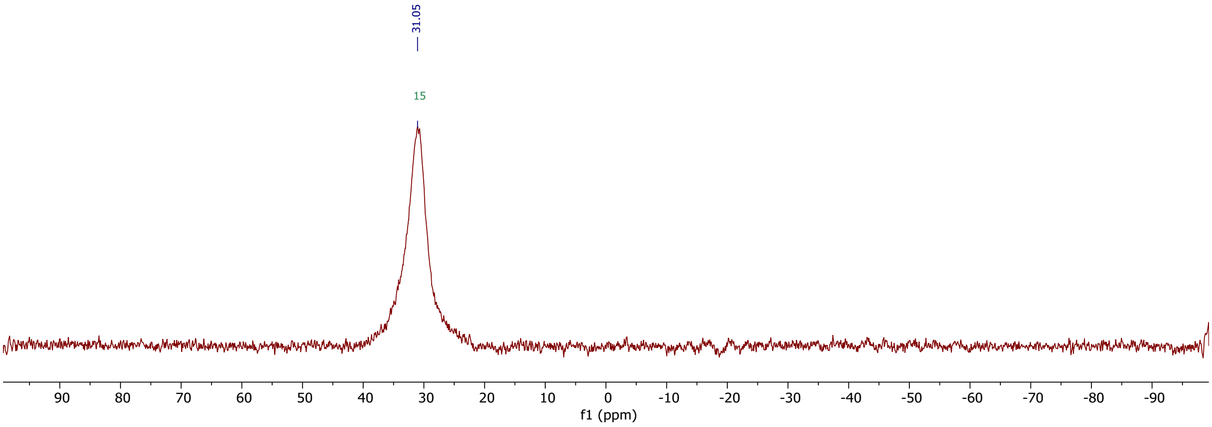

*Figure 17. ^{11}B-NMR of compound **9**.*

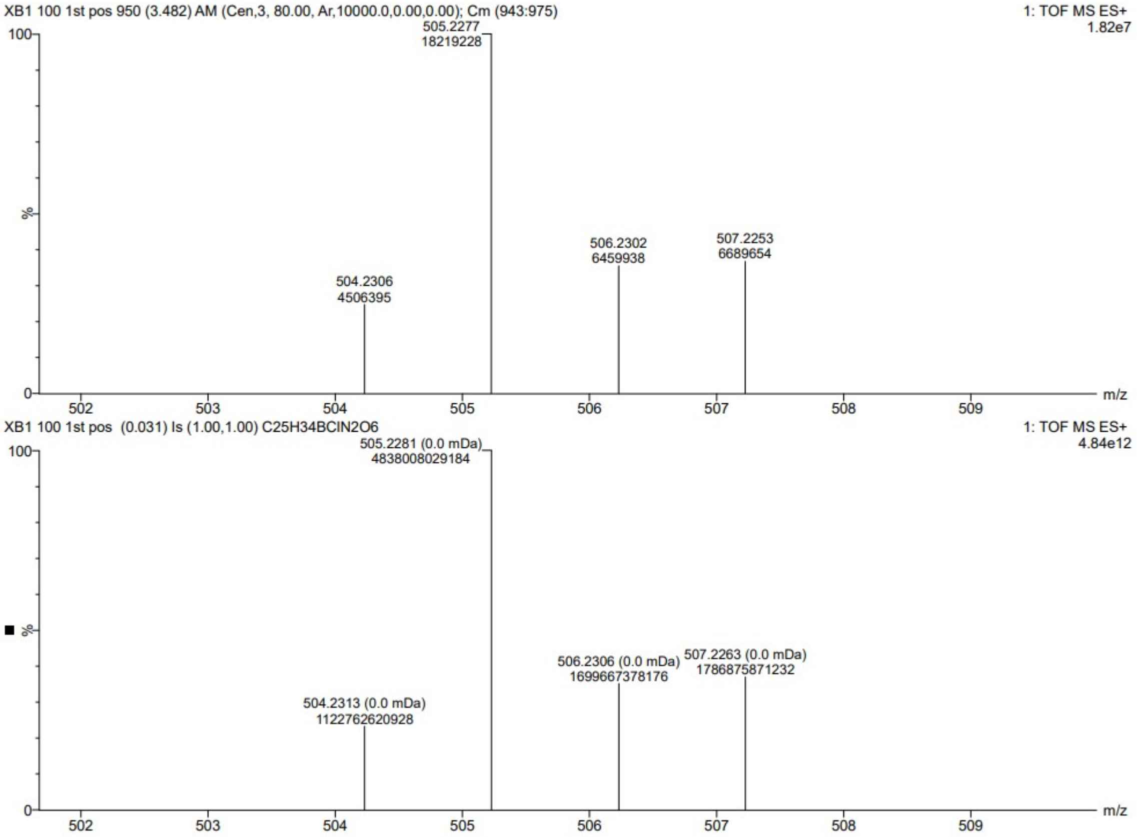

*Figure 18. HRMS of compound **9**.*

Compounds 10, 10A and (*S*)-10

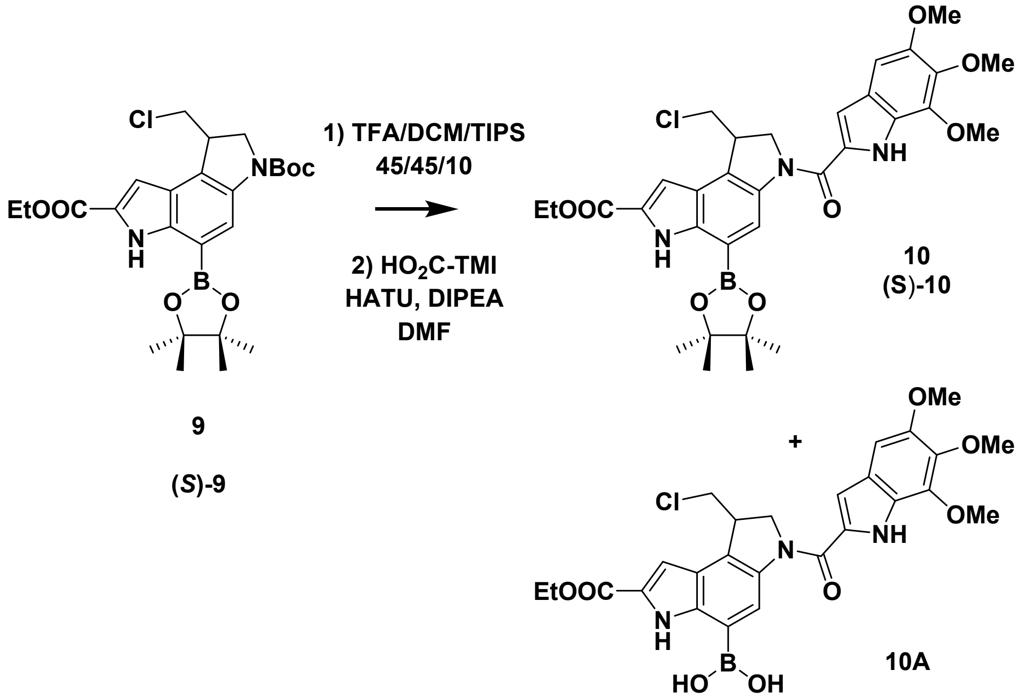

General method for the coupling of either 9 or (S)-9 to TMI-COOH.

Compound **9** or (*S*)-**9** (approx.. 100 mg, 1 eq) was deprotected by stirring for three hours in 5 mL of a solution 45/45/10 of TFA/CH_2Cl_2/TIPS. The solvent was removed under a flow of nitrogen to give a solid residue, which was used directly in the following step. HATU (1.1 eq) was added to a solution of TMI-COOH (1.1 eq) in 0.5 mL of DMF and fully dissolved before addition of DIPEA (2 eq). The resulting solution was mixed for 10 seconds and added to a stirring solution of the residue and DIPEA (4 eq) in 0.5 mL of DMF. After 18 hours of stirring, water was added to the solution dropwise, as much as possible but not enough to cause precipitation. The resulting solution was injected on 2 stacked Biotage 12 g C18 columns and eluted for 3 CV using Water + 0.05 % TFA, followed by a gradient up to 100 % acetonitrile + 0.05 % TFA in 40 CV; the elution was maintained at 100 % acetonitrile + 0.05 % TFA until complete elution of the product. Collected fractions with pure product were dried under reduced pressure.

Data for 10 and (S)-10

Data for **10** and (*S*)-**10** match except for specific rotation. While **10** does not show any optical activity, (*S*)-**10** has α_D^{25} -19 (c = 0.52 mg/mL, $CHCl_3$)

Yield of **10**: 48%

Yield of **(*S*)-10**: 33%

HRMS: $[M+H]^+$ m/z = 638.2496 (calculated = 638.2446)

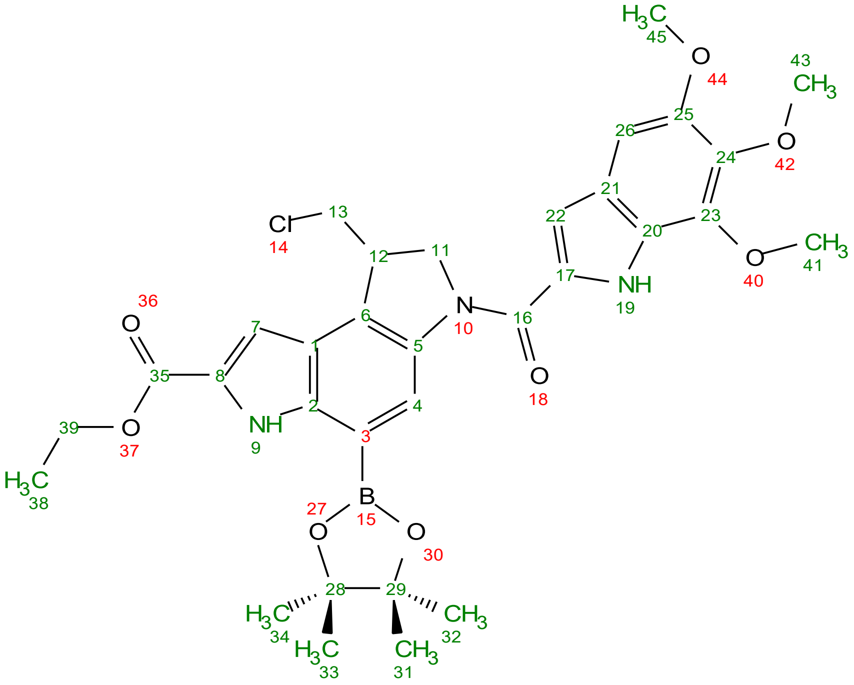

^{1}H NMR (400 MHz, Chloroform-d) δ 9.81 (s, 1H, 9), 9.49 (s, 1H, 19), 8.86 (s, 1H, 4), 7.16 (d, J = 2.1 Hz, 1H, 7), 6.97 (d, J = 2.3 Hz, 1H, 22), 6.88 (s, 1H, 26), 4.71 (dd, J = 10.8, 8.9 Hz, 1H, 11’), 4.63 (dd, J = 10.8, 4.1 Hz, 1H, 11’’), 4.44 (q, J = 7.1 Hz, 2H, 39), 4.18 (m, 1H, 12), 4.12 – 4.05 (m, 4H, 13’, 41), 3.95 (s, 3H, 43), 3.92 (s, 3H, 45), 3.64 (dd, J = 11.1, 9.7 Hz, 1H, 13’), 1.45 (t, J = 7.1 Hz, 3H, 38), 1.40 (s, 12H, 31, 32, 33, 34).

^{13}C NMR (101 MHz, CDCl3) δ 161.79 (35), 159.95 (16), 150.24 (25), 140.63 (24), 140.15 (2), 139.05 (23), 137.84 (5), 130.24 (17), 129.11 (8), 126.48 (6), 125.57 (20), 123.85 (21), 123.70 (4), 122.82 (1), 106.27 (22), 104.85 (7), 97.84 (26), 84.45 (28,29), 61.64 (43), 61.33 (39), 61.27 (41), 56.46 (45), 54.54 (11), 46.29 (13), 44.34 (12), 25.13 (31, 32, 33, 34), 14.52 (38).

^{11}B NMR (128 MHz, CDCl3) δ 31.31.

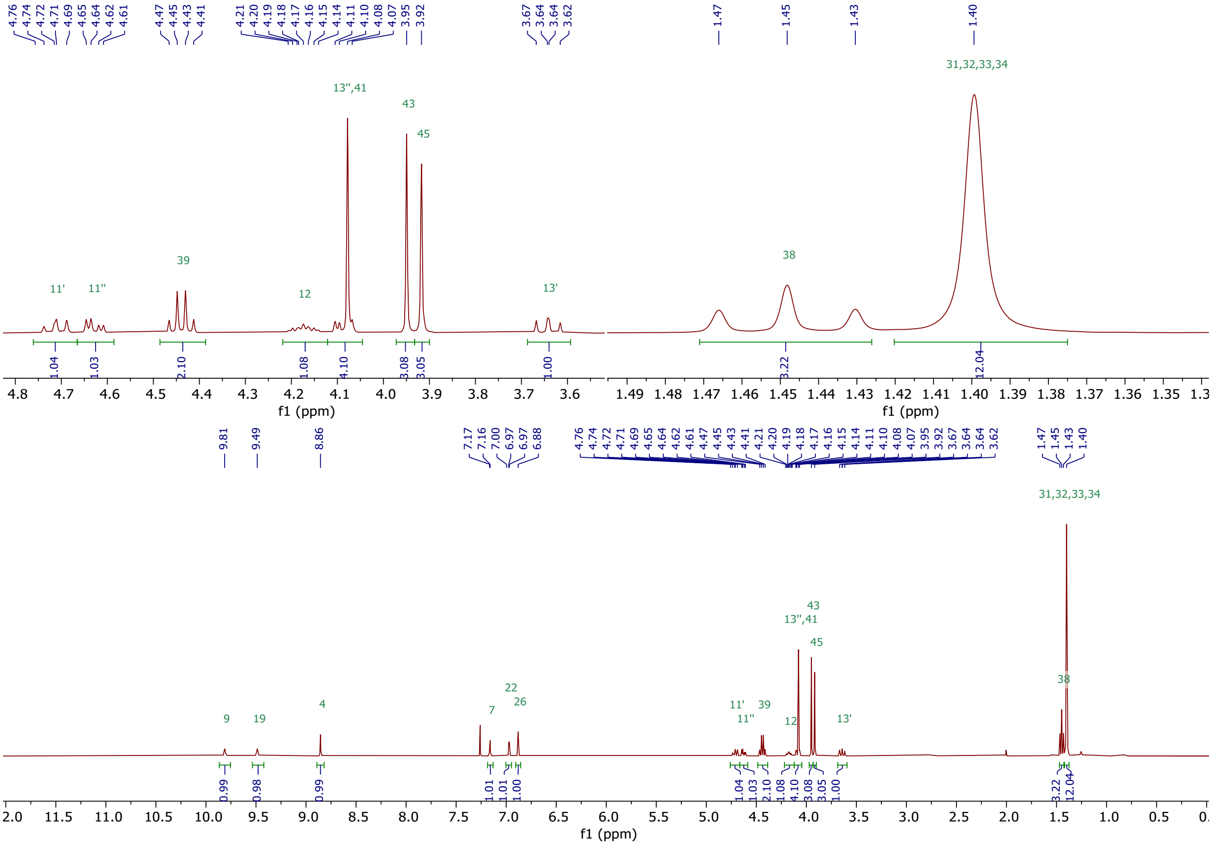

*Figure 19. ^{1}H-NMR of **10** and zoom in on regions of interest.*

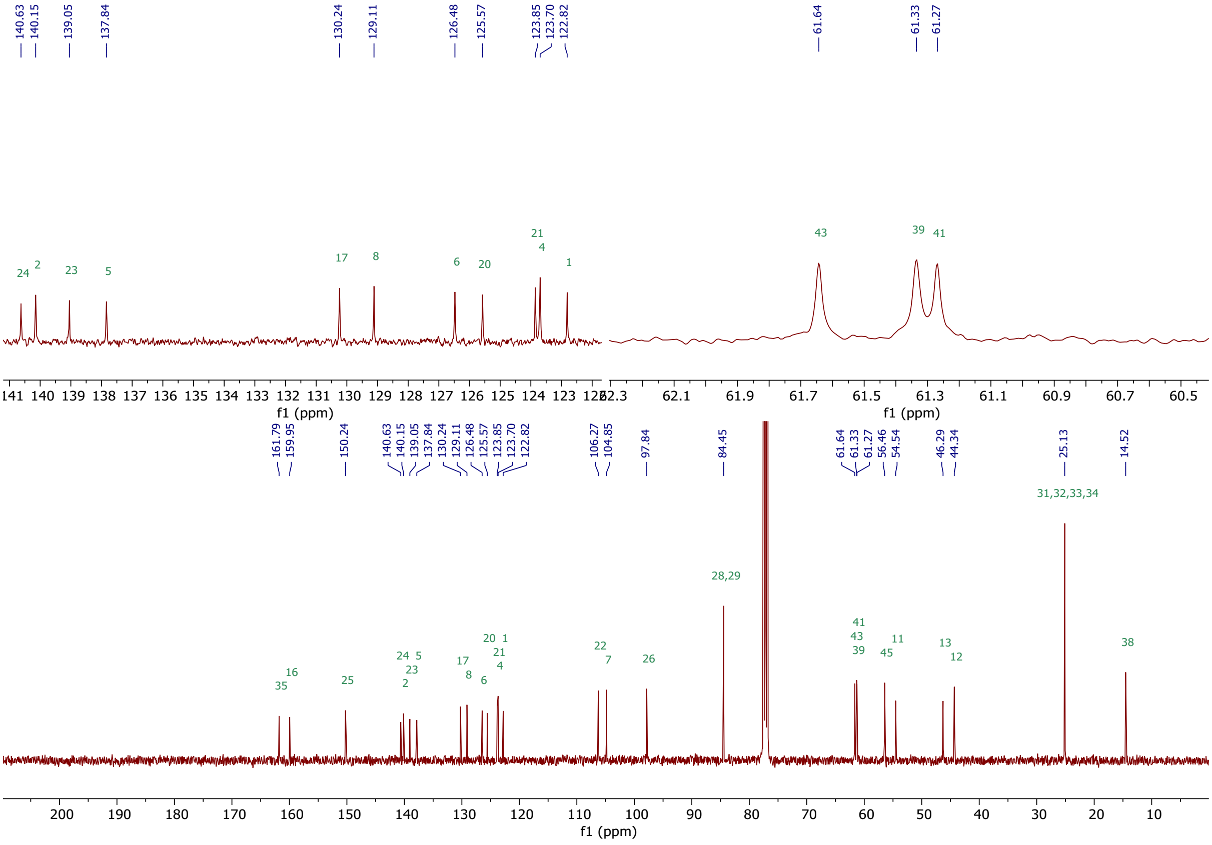

*Figure 20. ^{13}C-NMR of **10** and zoom in on regions of interest.*

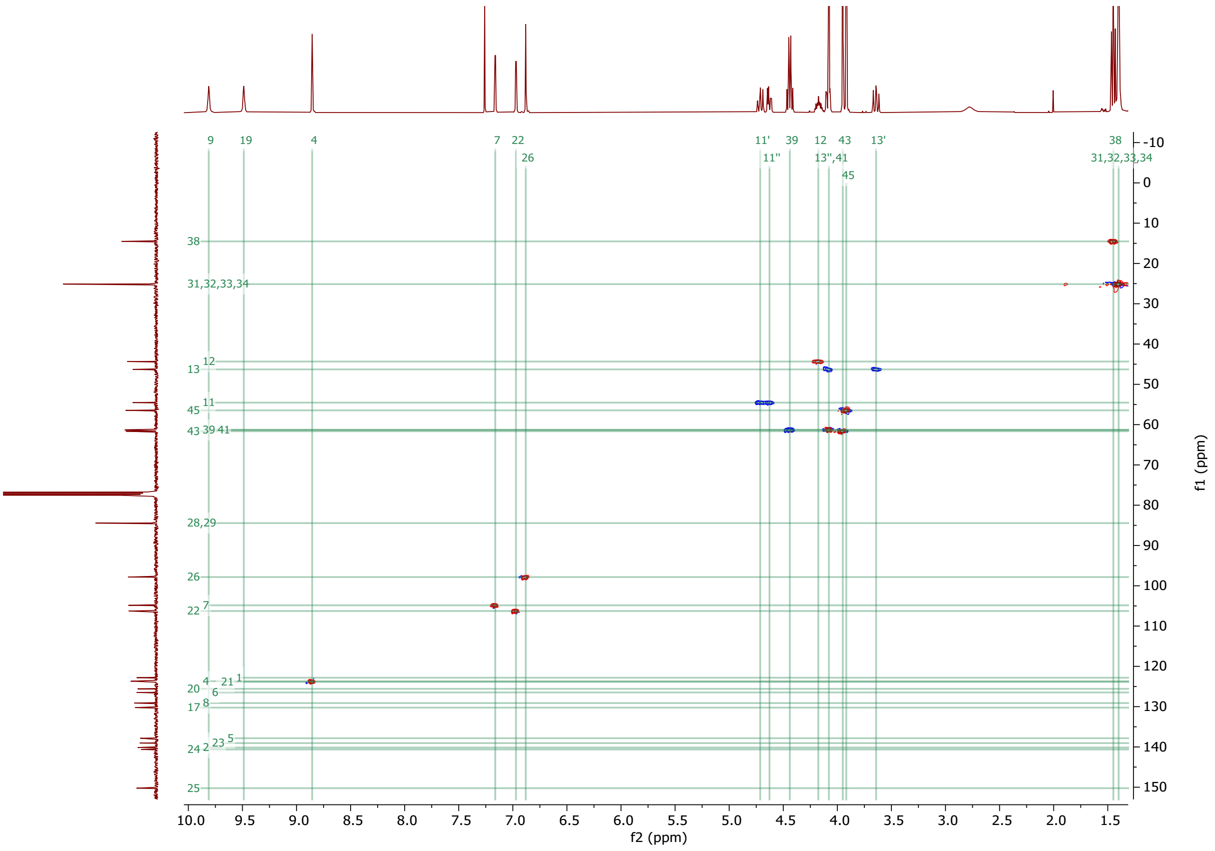

Figure 21. HSQC of compound **10**.

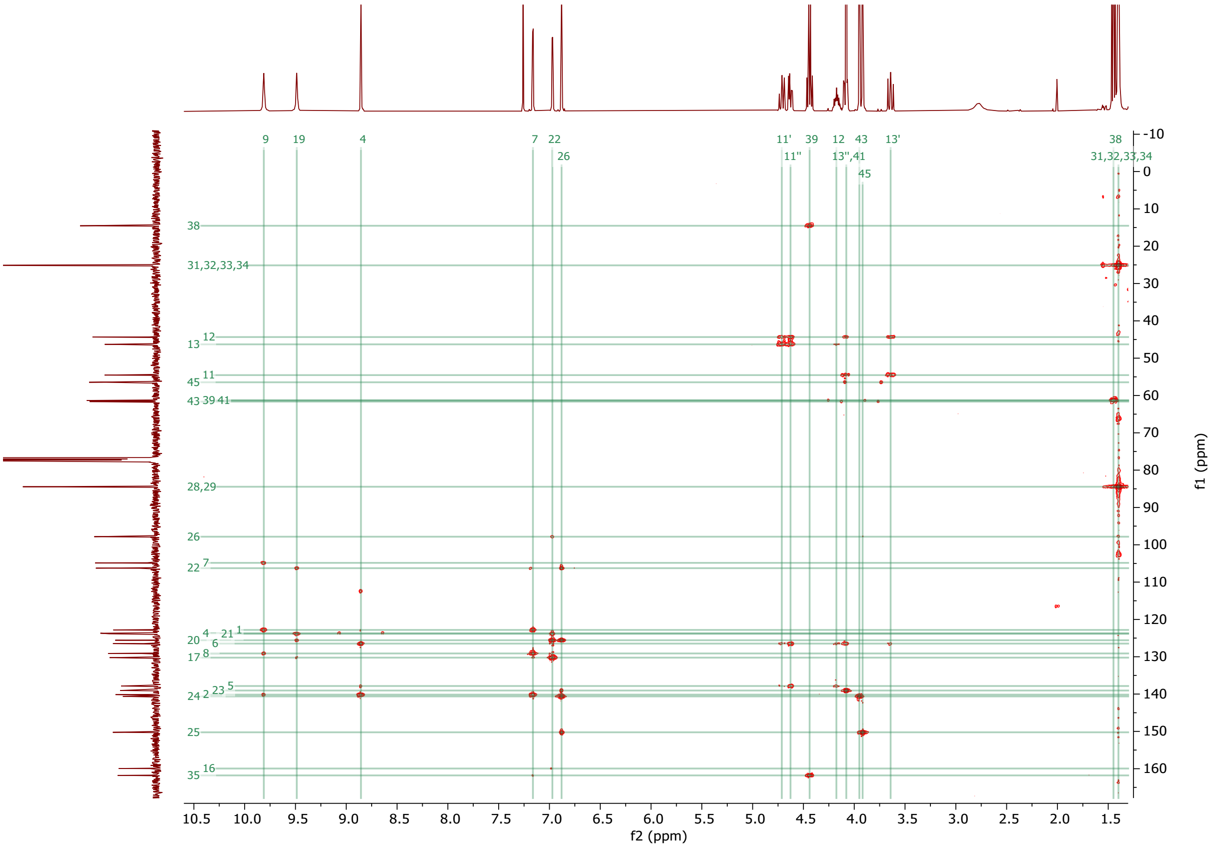

Figure 22. HMBC of compound **10**.

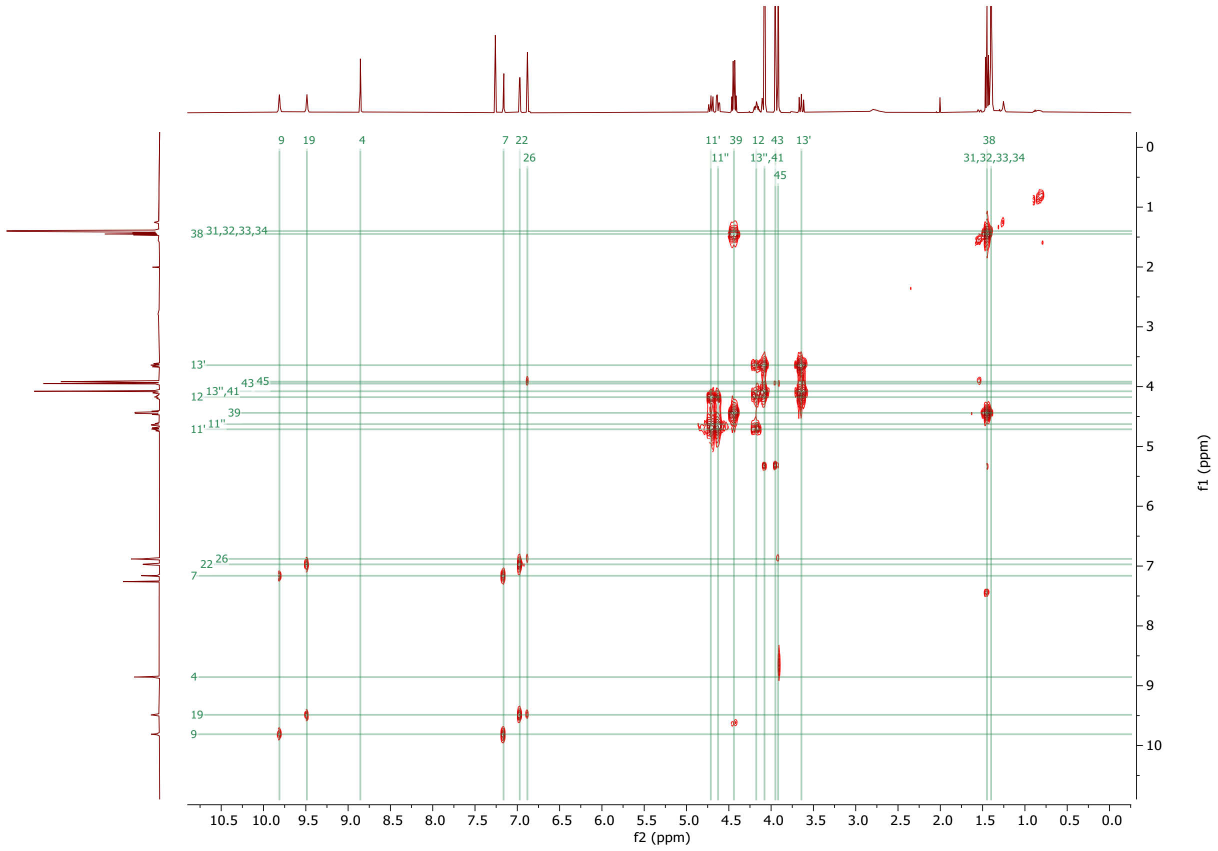

*Figure 23. COSY of compound **10**.*

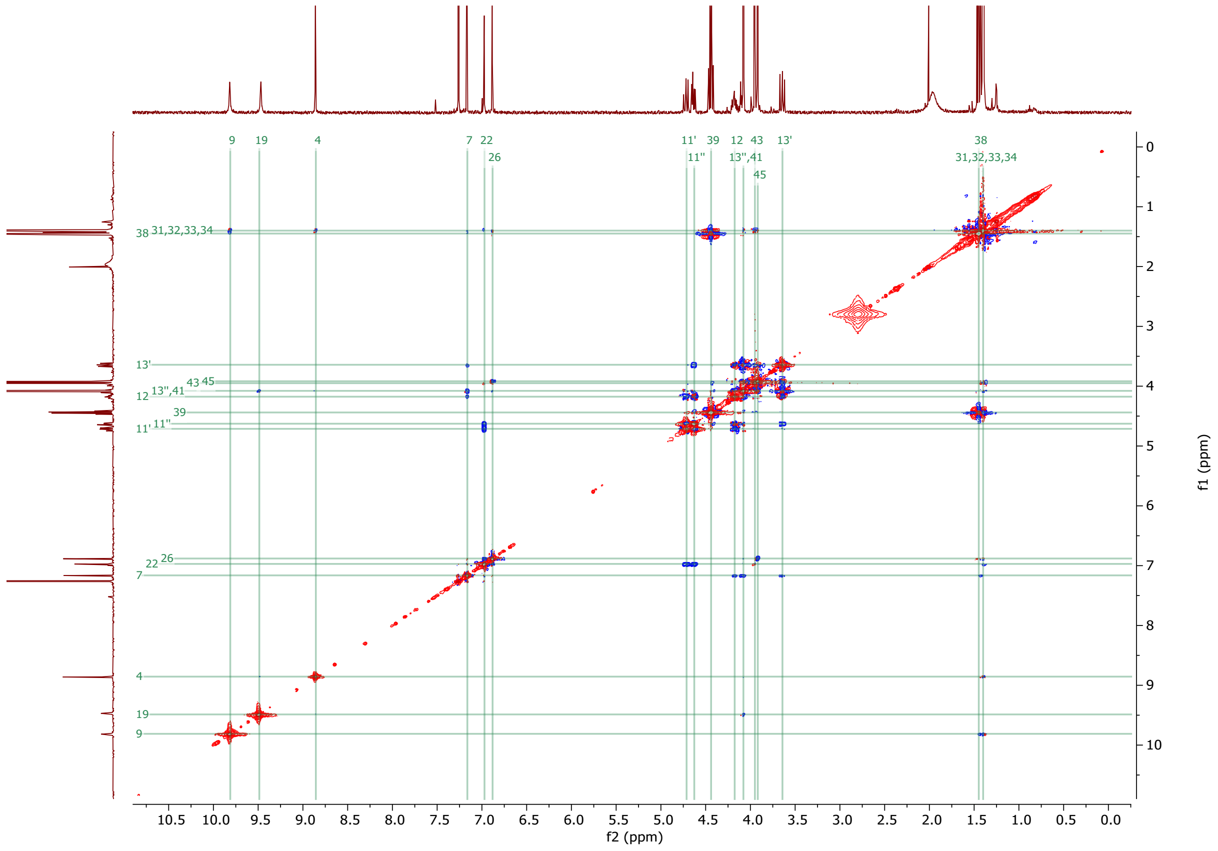

*Figure 24. NOESY of compound **10**.*

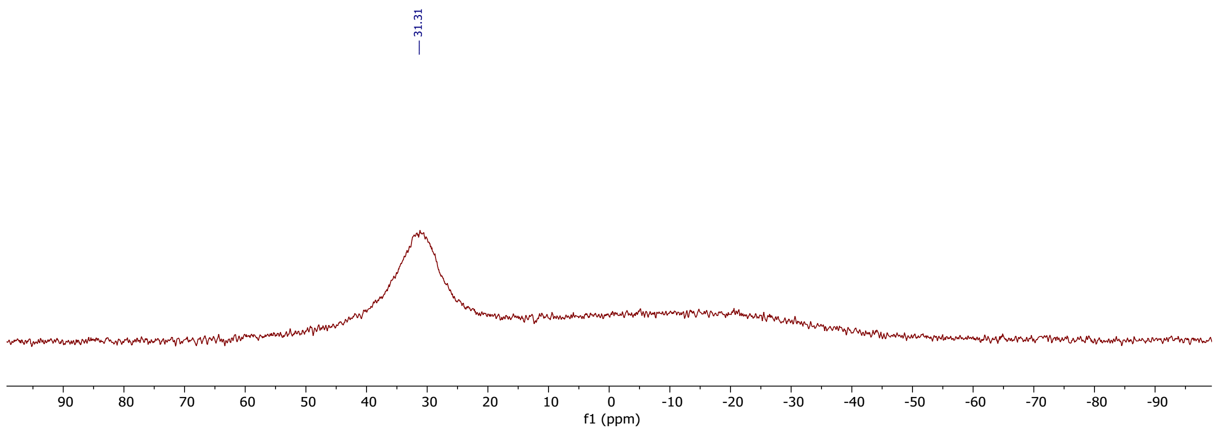

*Figure 25. 11B-NMR of compound **10**.*

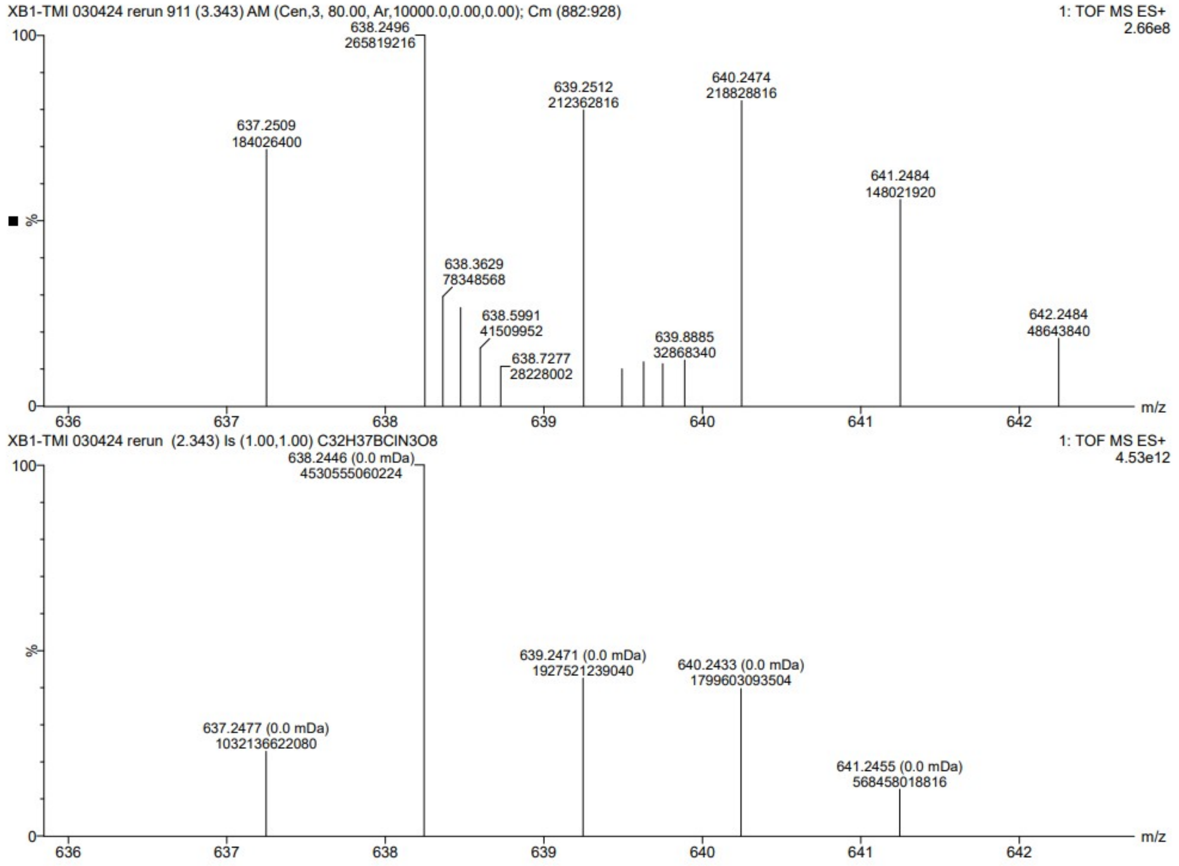

*Figure 26. HRMS of **10**.*

Data for 10A

Yield of **10A**: 33%

HRMS: $[M+H]^+$ m/z = 556.1663 (calculated = 556.1663)

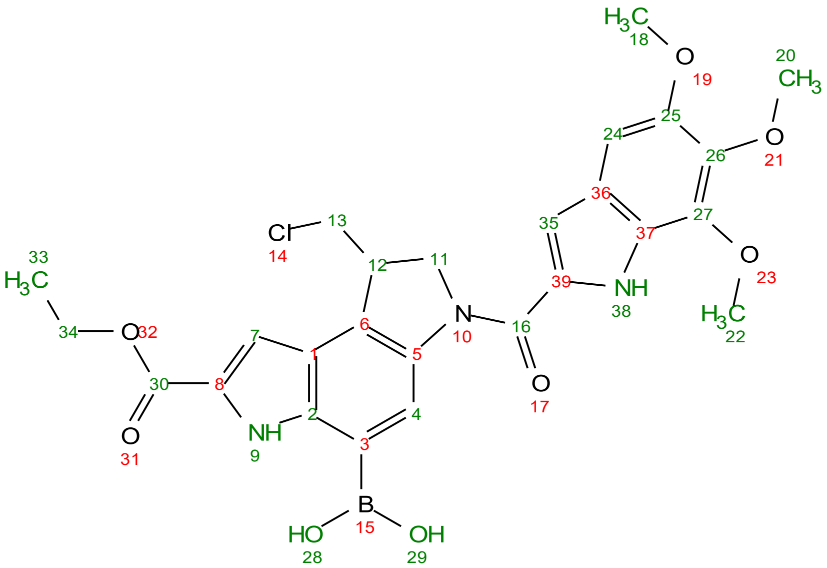

^{1}H-NMR (400 MHz, Acetone-d_6) δ 10.35 (bs, 1H, 9), 10.25 (bs, 1H, 38), 8.81 (s, 1H, 4), 8.06 (bs, 2H, 28, 29), 7.34 (d, *J* = 2.3 Hz, 1H, 7), 7.10 (d, *J* = 2.3 Hz, 1H, 35), 6.99 (s, 1H, 24), 4.82 (dd, *J* = 10.8, 9.3 Hz, 1H, 11’), 4.63 (dd, *J* = 10.9, 4.3 Hz, 1H, 11’’), 4.44 – 4.37 (m, 2H, 34), 4.36 – 4.29 (m, 1H, 12), 4.23 (dd, *J* = 11.1, 3.7 Hz, 1H, 13’’), 4.03 (s, 3H,), 4.02 (dd, J = 11.1, 7.7 Hz, 1H, 13’), 3.88 (s, 3H, 18), 3.87 (s, 3H, 20), 1.39 (t, *J* = 7.1 Hz, 3H, 33).

^{13}C-APT-NMR (101 MHz, Acetone) δ 161.20 (30), 159.61 (16), 150.12 (25), 140.20 (26), 139.98 (HMBC, 2), 139.11 (27), 138.20, 131.21, 128.42, 126.33, 123.93, 122.99, 122.82 (4), 105.91 (35), 104.77 (7), 98.19 (24), 60.59 (34), 60.53 (20 or 22), 60.52 (20 or 22), 55.65 (18), 54.39 (11), 46.70 (13), 43.33 (12), 13.79 (33).

^{11}B-NMR (128 MHz, Acetone) δ 29.17.

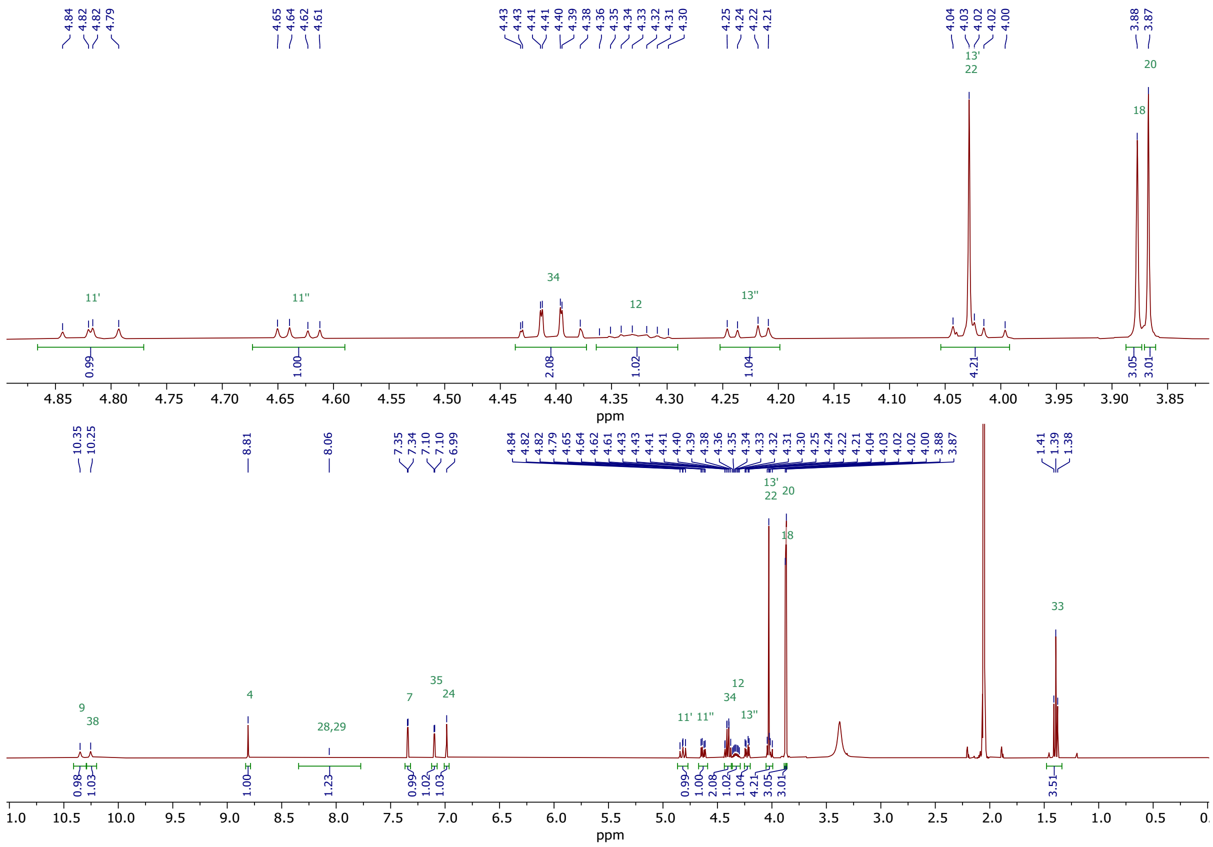

*Figure 27. ^{1}H-NMR of **10A** and zoom in on regions of interest.*

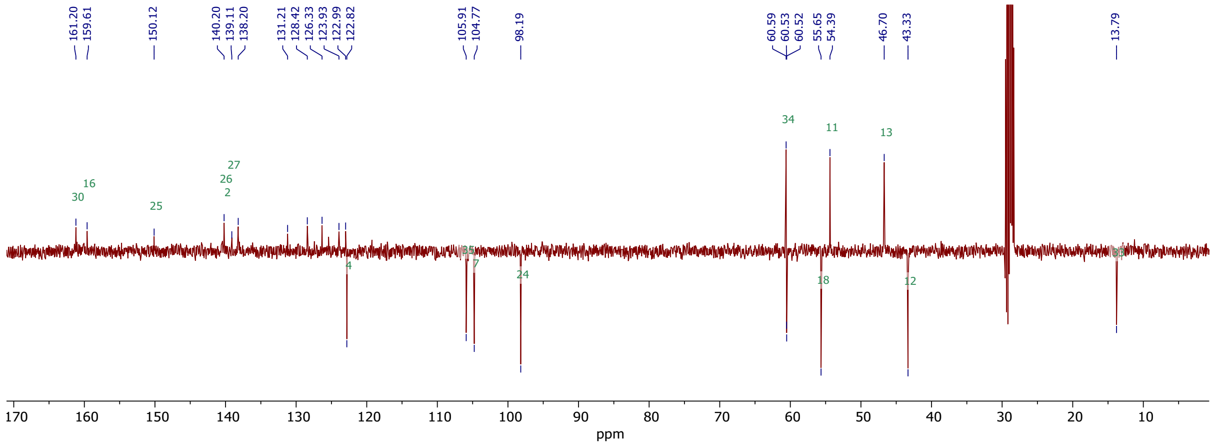

*Figure 28. ^{13}C-APT of **10A**.*

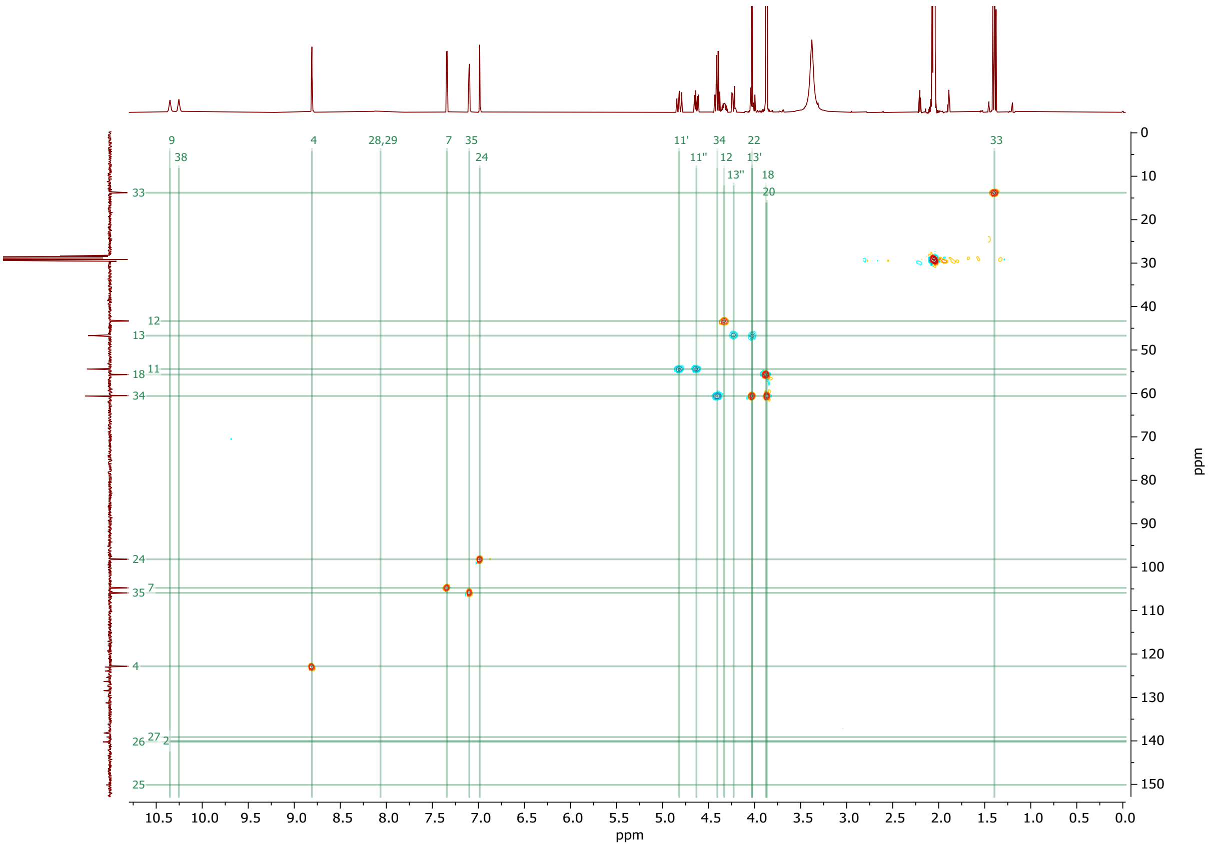

*Figure 29. HSQC of compound **10A**.*

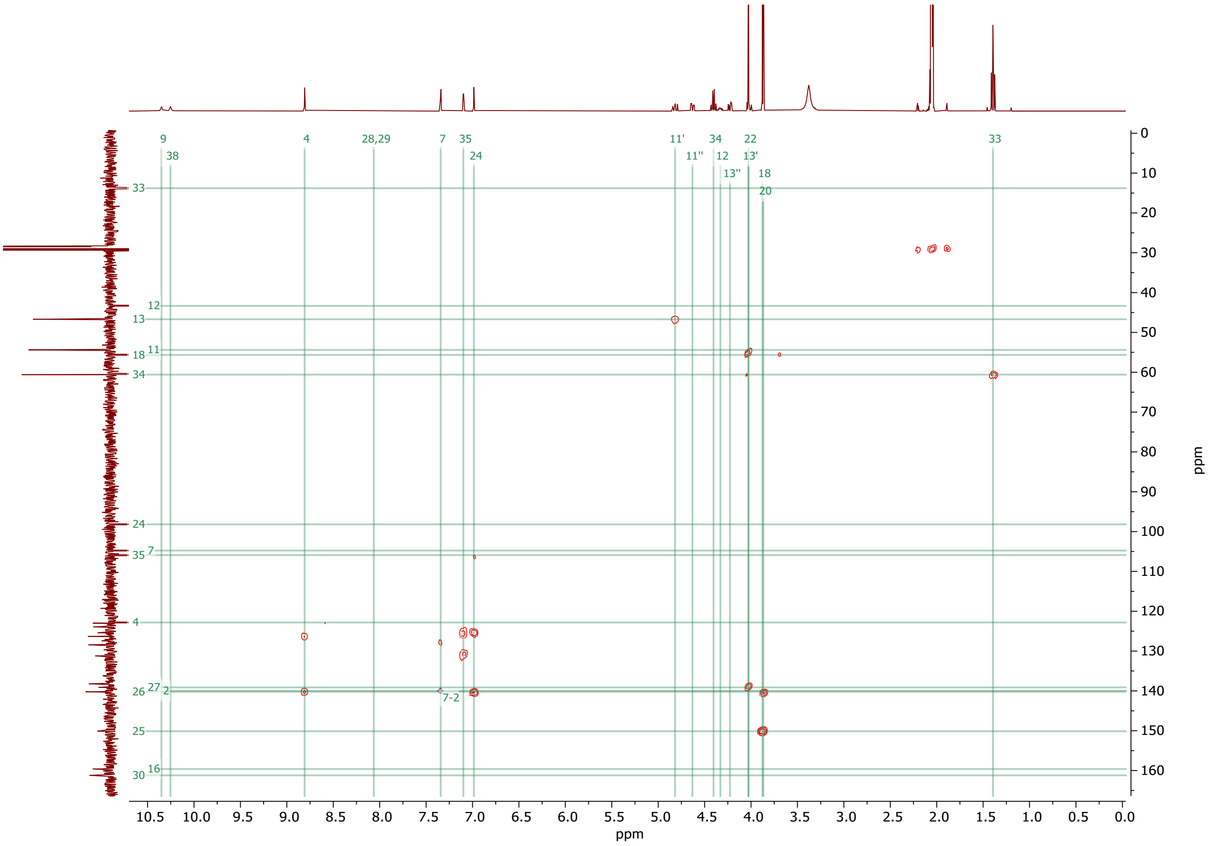

*Figure 30. HMBC of compound **10A**.*

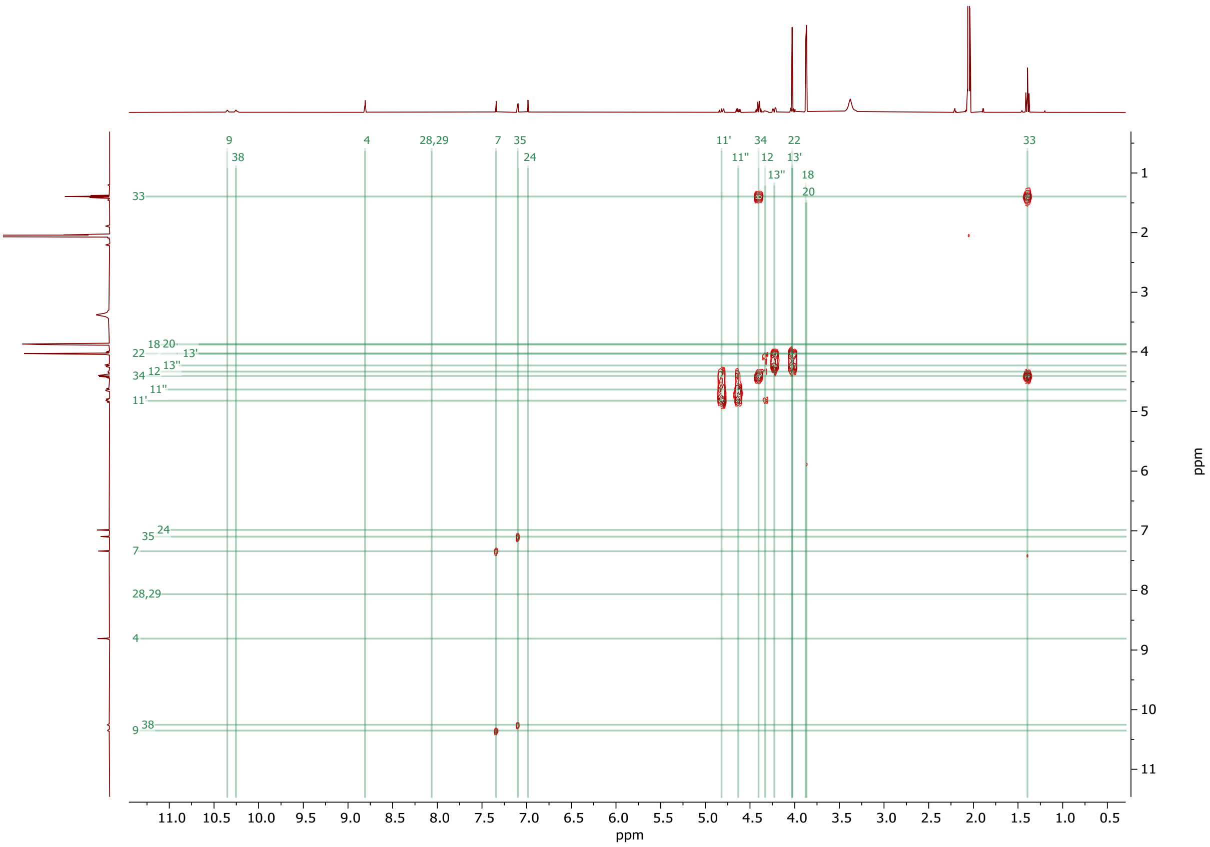

*Figure 31. COSY of compound **10A**.*

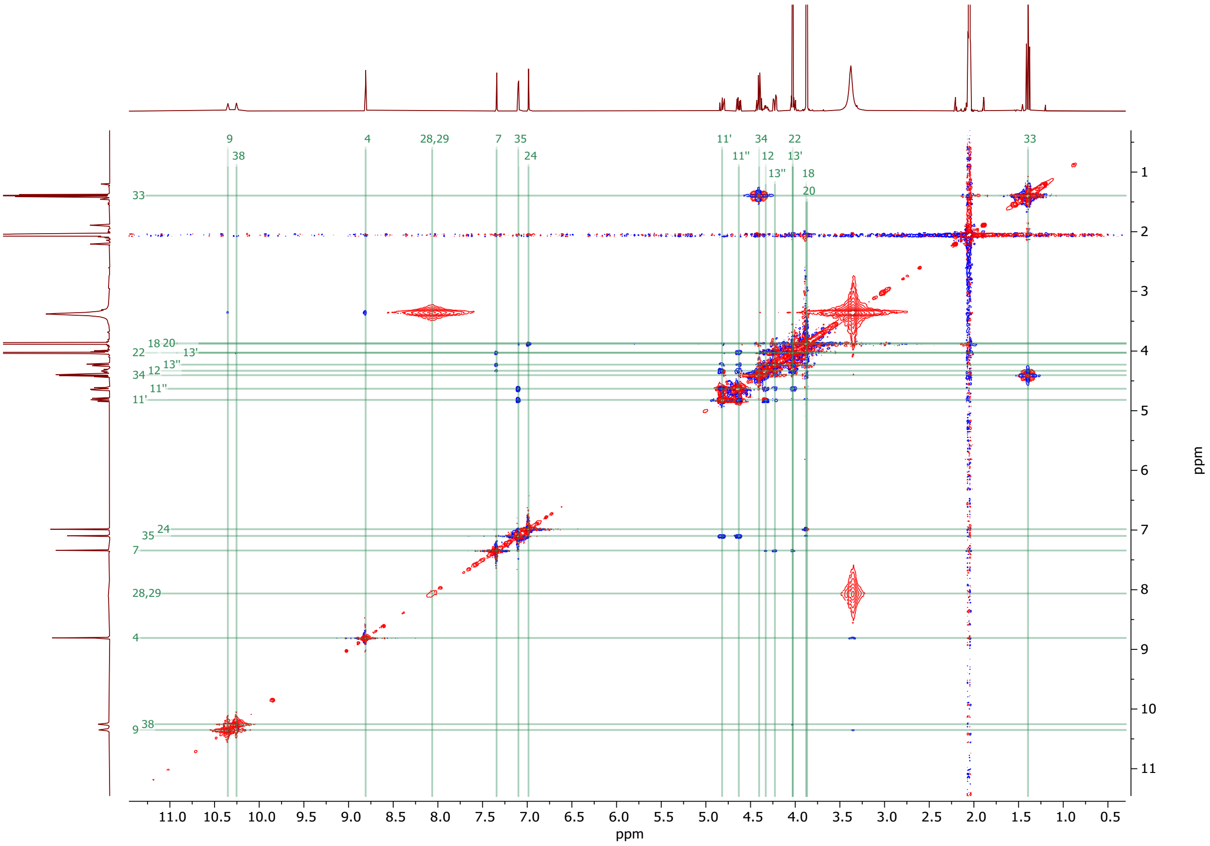

*Figure 32. NOESY of compound **10A**.*

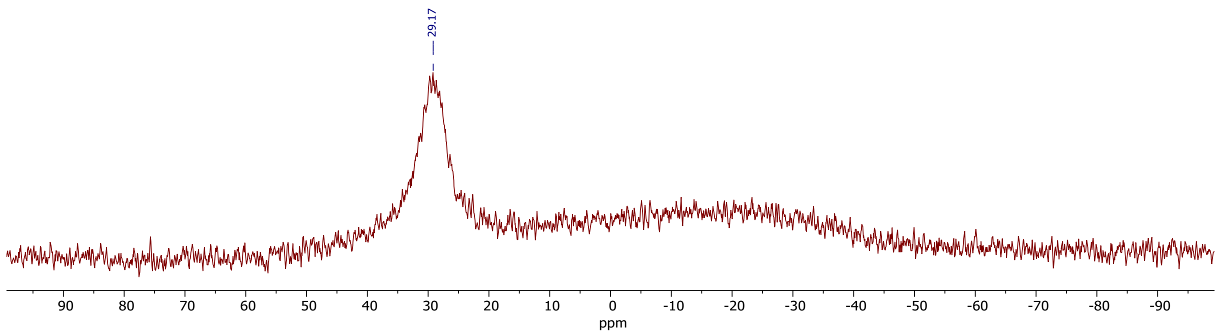

*Figure 33. 11B-NMR of compound **10A**.*

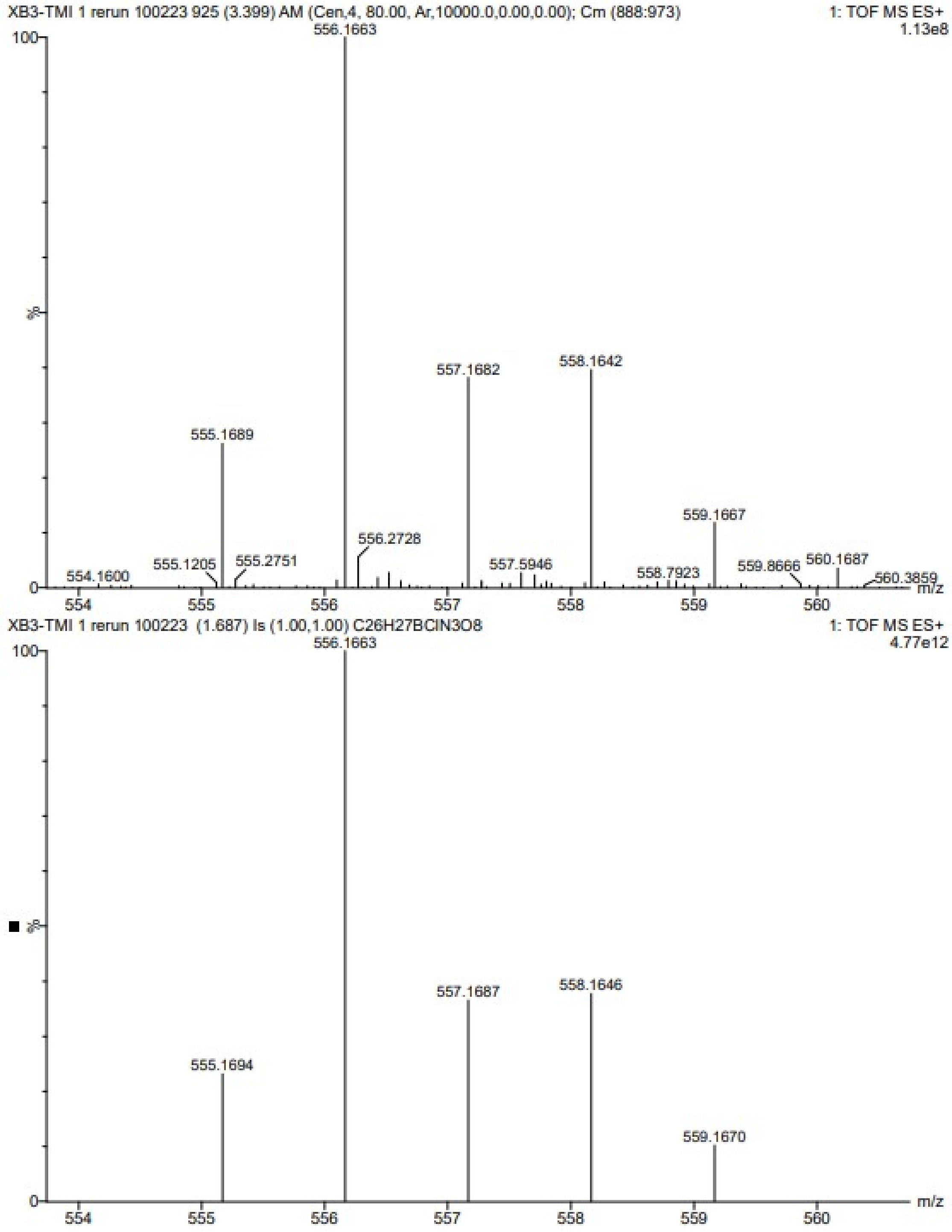

*Figure 34. HRMS of **10A**.*

Compound 4

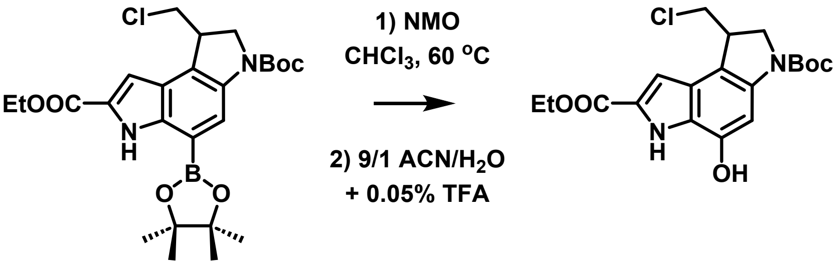

A solution of **9** (87 mg, 0.17 mmol) in 2.4 mL of chloroform is stirred at 60 °C for 20 hours. Reaction completion is assessed by ^{11}B-NMR and the solvent is removed under reduced pressure. The solid residue is dissolved in 1.5 mL of 9/1 acetonitrile/water with 0.05% TFA and directly injected on two stacked Biotage 12 g C18 columns and eluted for 3 CV with 100% water + 0.05% TFA, followed by a linear gradient to 100% of acetonitrile + 0.05% TFA over 40 CV. Fractions containing the product were collected and freeze dried to give **4** (61.1 mg) as a green-brown solid.

Yield of **4**: 90%

HRMS: $[M+H]^+$ m/z = 395.1377 (calculated = 395.1374)

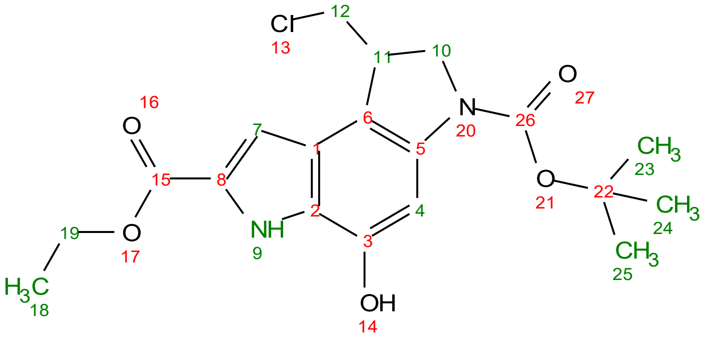

^{1}H NMR (400 MHz, Acetone-d6) δ 10.61 (s, 1H, 9), 7.59 (bs, 1H, 4), 7.18 (d, J = 2.0 Hz, 1H, 7), 4.35 (qd, J = 7.1, 0.6 Hz, 2H, 19), 4.19 – 4.12 (m, 1H, 10’’), 4.08 (dd, J = 10.9, 3.4 Hz, 1H, 12’’), 4.06 – 4.01 (m, 1H, 10’), 4.01 – 3.95 (m, 1H, 11), 3.76 (dd, J = 10.8, 8.3 Hz, 1H, 12’), 1.55 (s, 9H, 23, 24, 25), 1.36 (t, J = 7.1 Hz, 3H, 18).

^{13}C NMR (101 MHz, Acetone) δ 161.94 (15), 152.83 (26), 144.77, 129.54, 125.86, 106.23 (7), 99.84 (4), 80.48 (22), 61.26 (19), 53.49 (10), 48.15 (12), 28.64 (23, 24, 25), 14.66 (18).

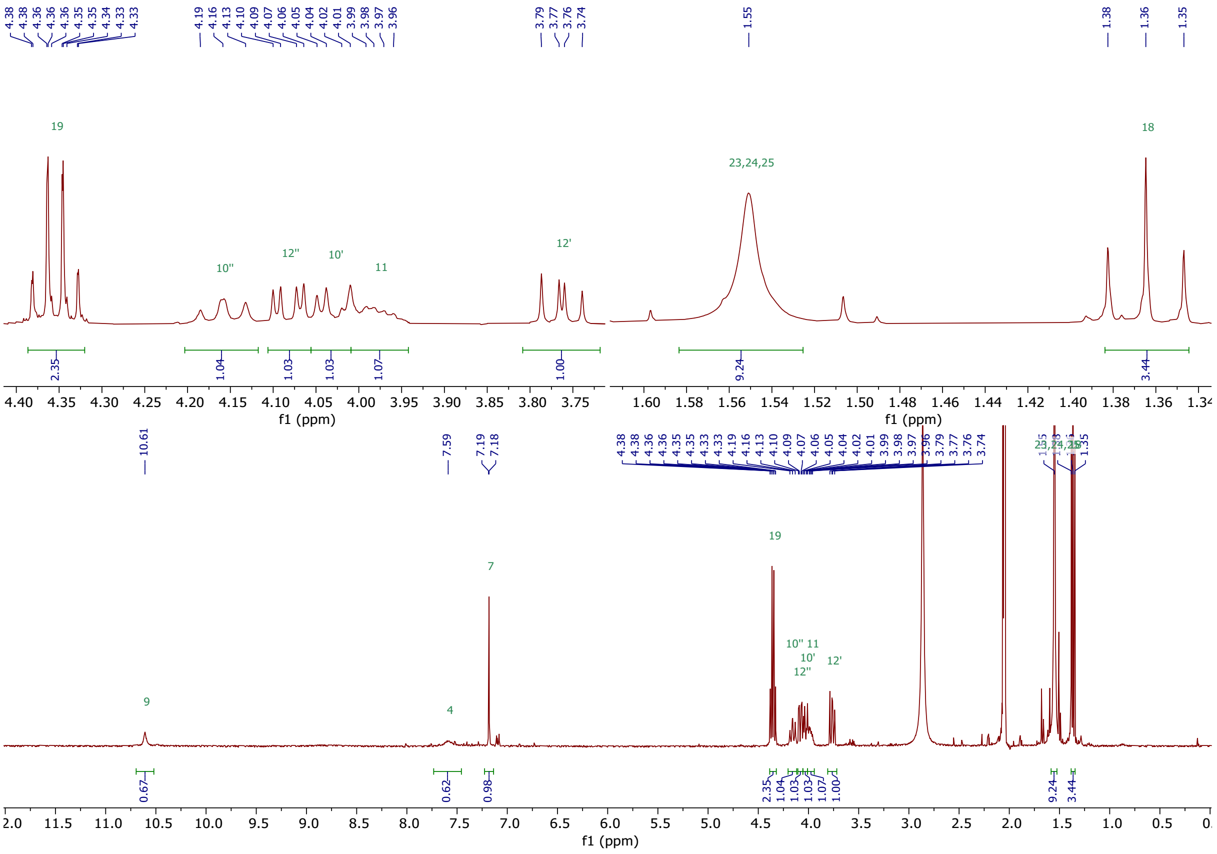

*Figure 35. ^{1}H-NMR of **4** and zoom in on regions of interest.*

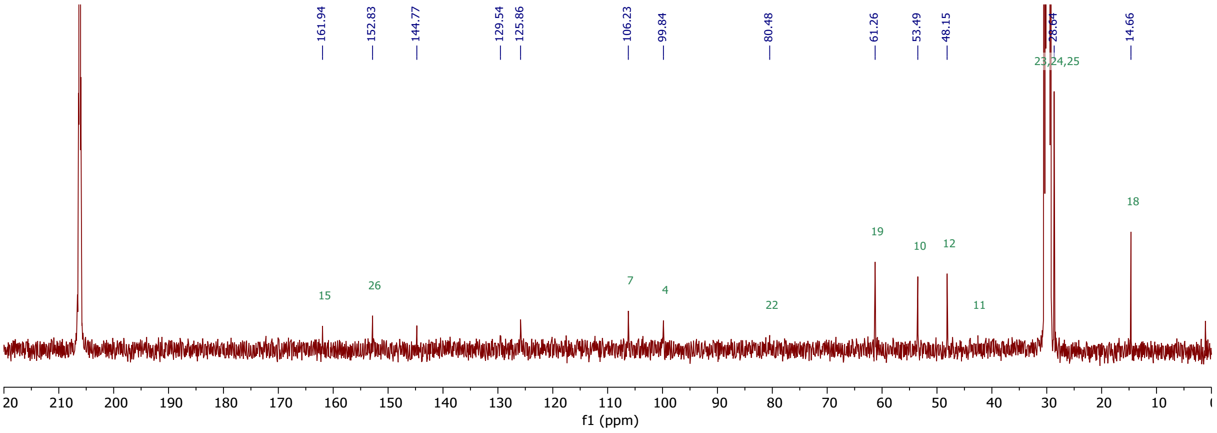

*Figure 36. ^{13}C-NMR of **4**.*

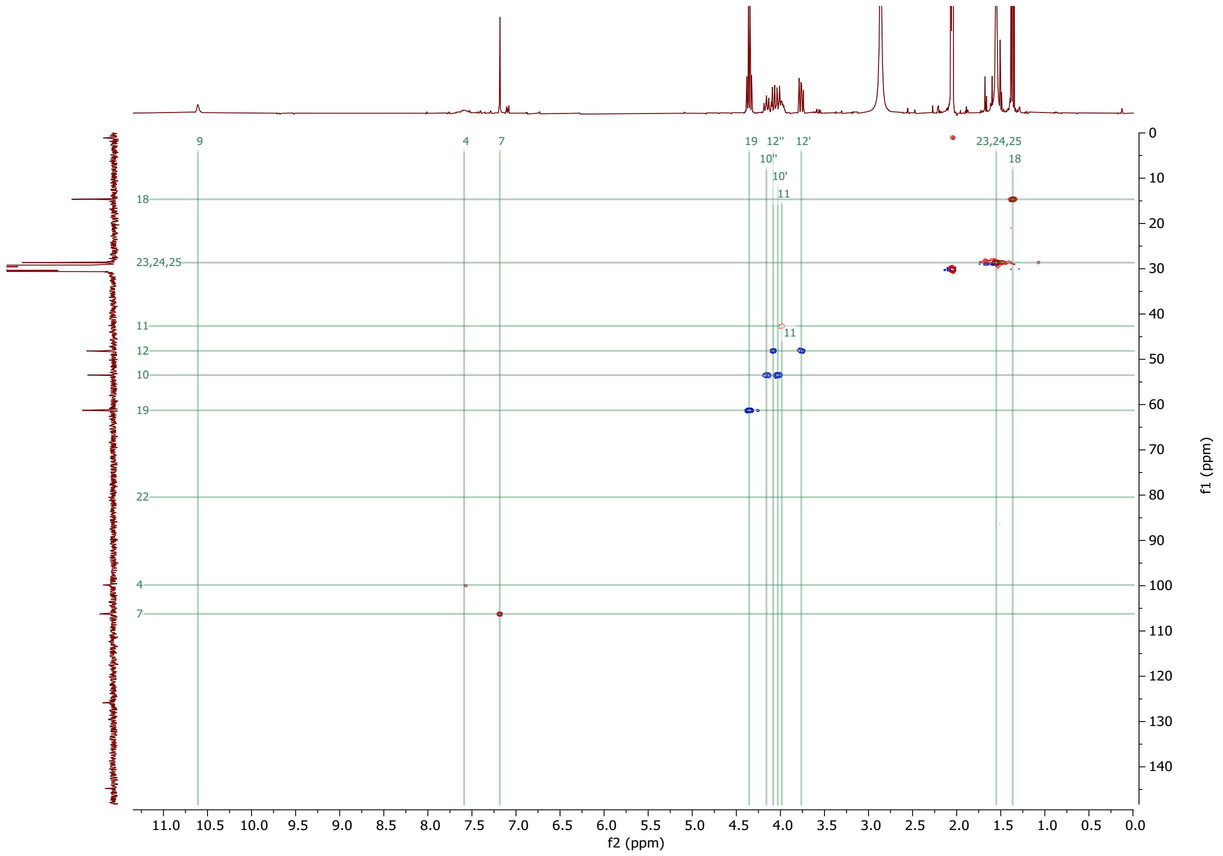

*Figure 37. HSQC of compound **4**.*

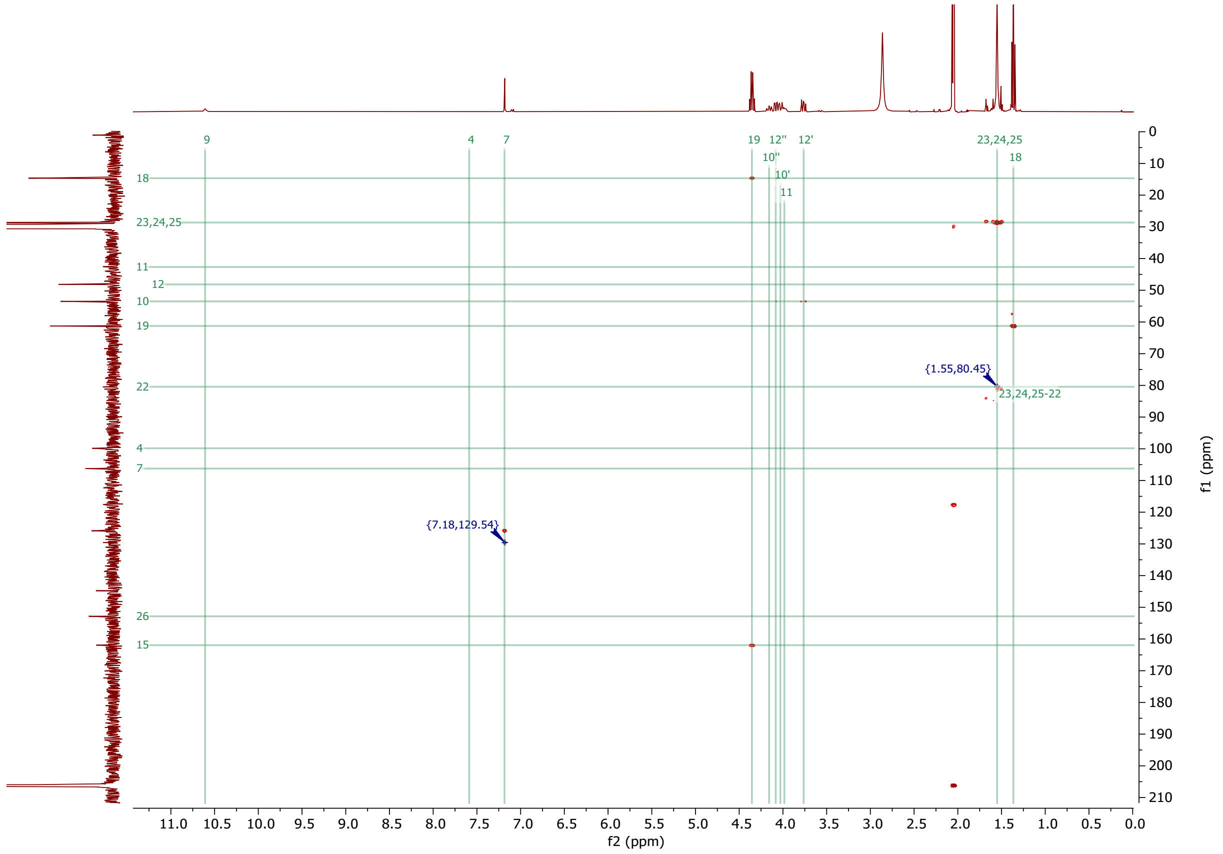

*Figure 38. HMBC of compound **4**.*

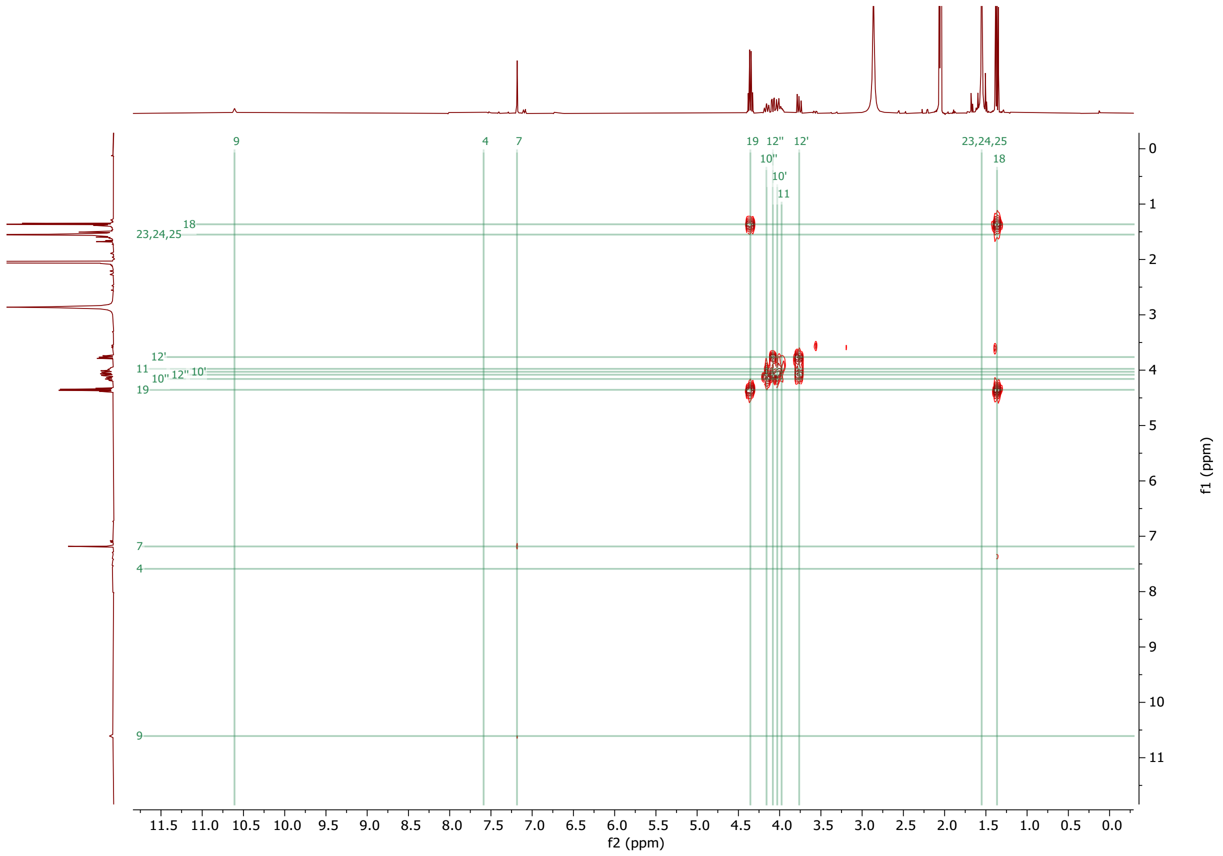

*Figure 39. COSY of compound **4**.*

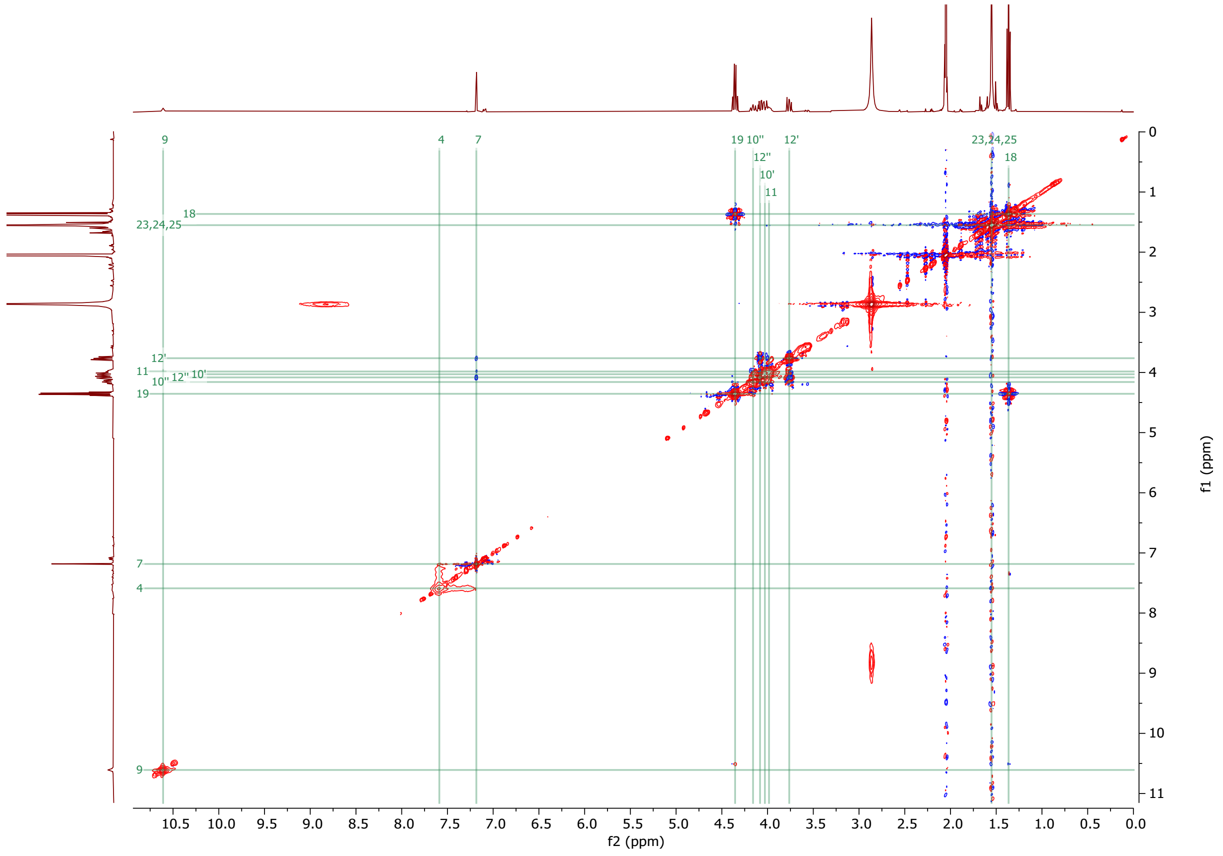

*Figure 40. NOESY of compound **4**.*

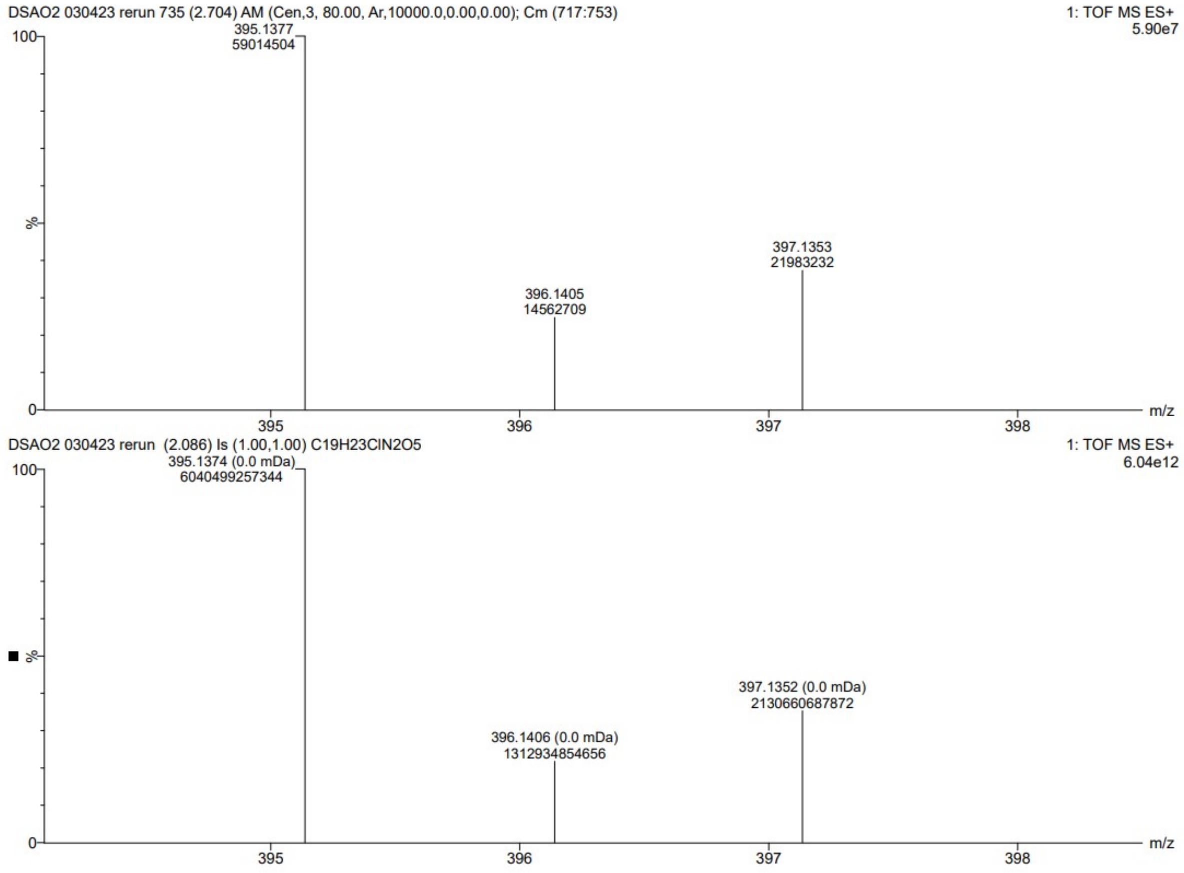

*Figure 41. HRMS of compound **4**.*

Compound 11 and (*S*)-11

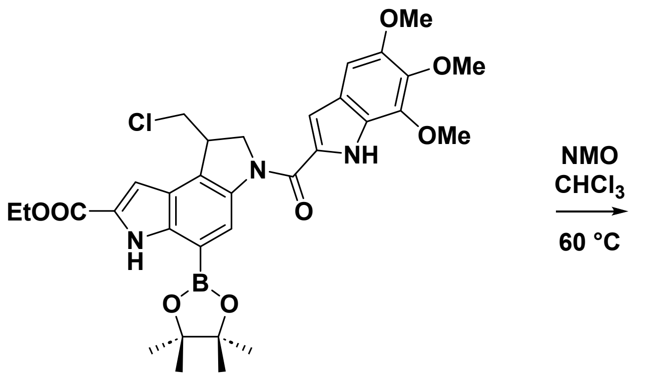

Both **11** and (*S*)-**11** are synthesised following the example method below.

A solution of **10** (17.05 mg, 0.027 mmol) and NMO (12.5 mg, 0.107 mmol) in 0.5 mL of chloroform is stirred at 60 °C for 20 hours. Reaction completion is assessed by ^{11}B-NMR and the solvent is removed under reduced pressure. The solid residue is dissolved in mL of 9/1 acetonitrile/water with 0.05% TFA and directly injected on C18 columns and eluted for 3 CV with 100% water + 0.05% TFA, followed by a linear gradient to 100% of acetonitrile + 0.05% TFA over 40 CV. Fractions containing the product were collected and dried to give **11** as an off-white solid (7.81 mg).

Yield of **11**: 55 %

Yield of (*S*)-**11**: 34%

Data for **11** and (*S*)-**11** match except for specific rotation. While **11** does not show any optical activity, (*S*)-**11** has α_D^{25} -16 (c = 0.61 mg/mL, THF)

HRMS: $[M+H]^+$ m/z = 528.1554 (calculated = 528.1537)

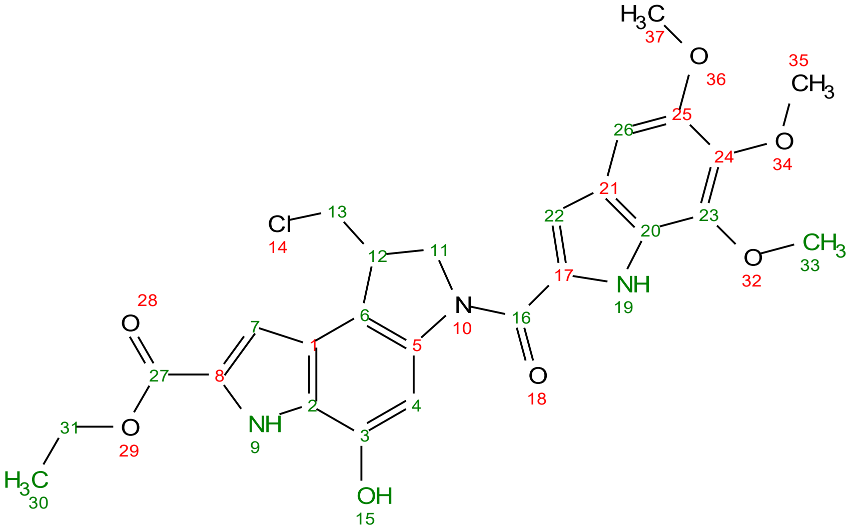

^{1}H NMR (400 MHz, DMSO-d6) δ 11.58 (d, *J* = 1.9 Hz, 1H, 9), 11.35 (d, *J* = 2.0 Hz, 1H, 19), 9.81 (s, 1H, 15), 7.73 (s, 1H, 4), 7.26 (d, *J* = 2.1 Hz, 1H, 7), 6.98 (d, *J* = 2.1 Hz, 1H, 22), 6.96 (s, 1H, 26), 4.67 (t, *J* = 10.1 Hz, 1H, 11'), 4.37 – 4.30 (m, 3H, 11'', 31), 4.12 – 4.01 (m, 2H, 13'', 12), 3.93 (s, 4H, 13', 33), 3.81 (s, 3H, 35 or 37), 3.79 (s, 3H, 35 or 37), 1.35 (t, *J* = 7.1 Hz, 3H, 30).

^{13}C NMR (101 MHz, DMSO-d6) δ. 160.99 (27), 159.55 (16), 149.08, 143.55 (3), 139.64, 139.05 (23), 138.20, 131.47, 128.16, 125.69, 125.06 (2), 123.93 (20), 123.20, 112.84 (6), 106.07 (7), 105.50 (2), 100.33 (4), 98.01 (26), 61.10 (33), 60.94, 61.47 (31), 55.96, 54.76 (11), 47.50 (13), 41.83 (12), 14.29 (30).

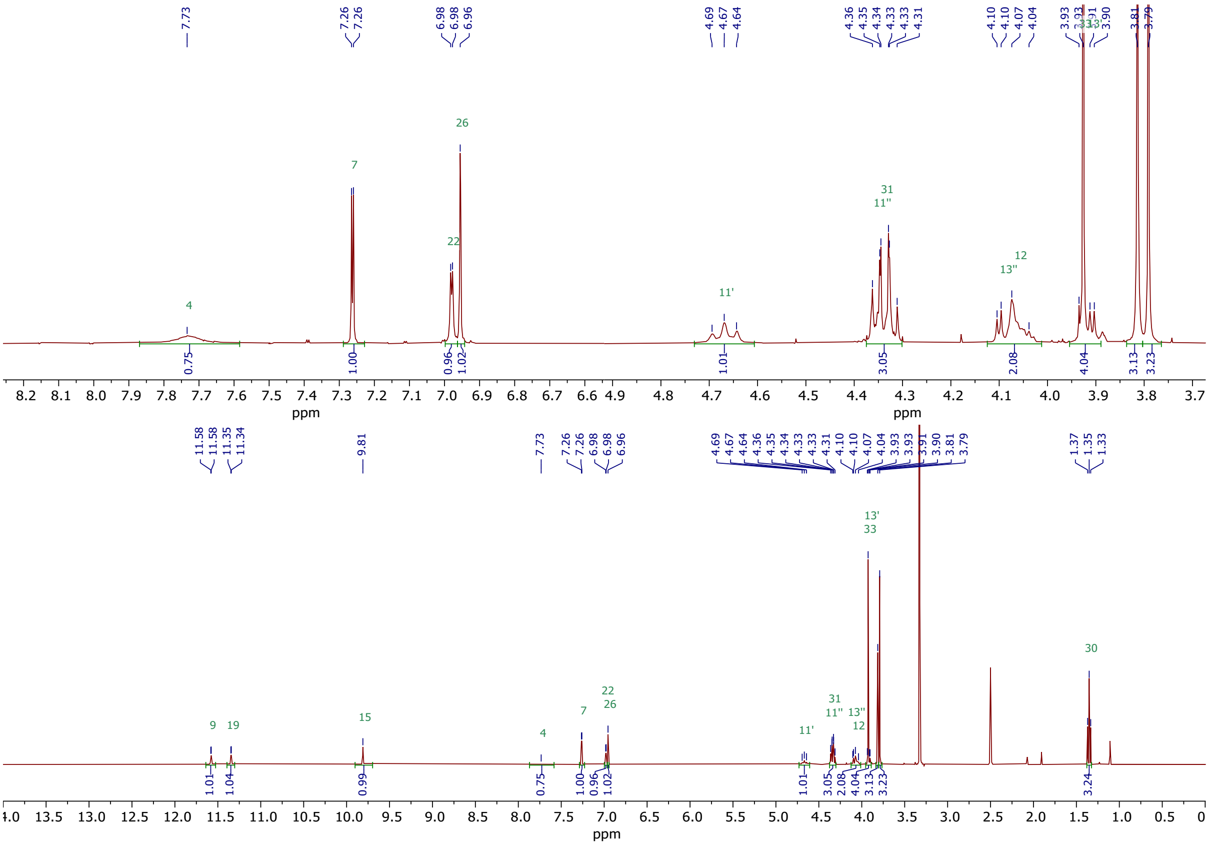

Figure 42. ¹H-NMR of **11** and zoom in on regions of interest.

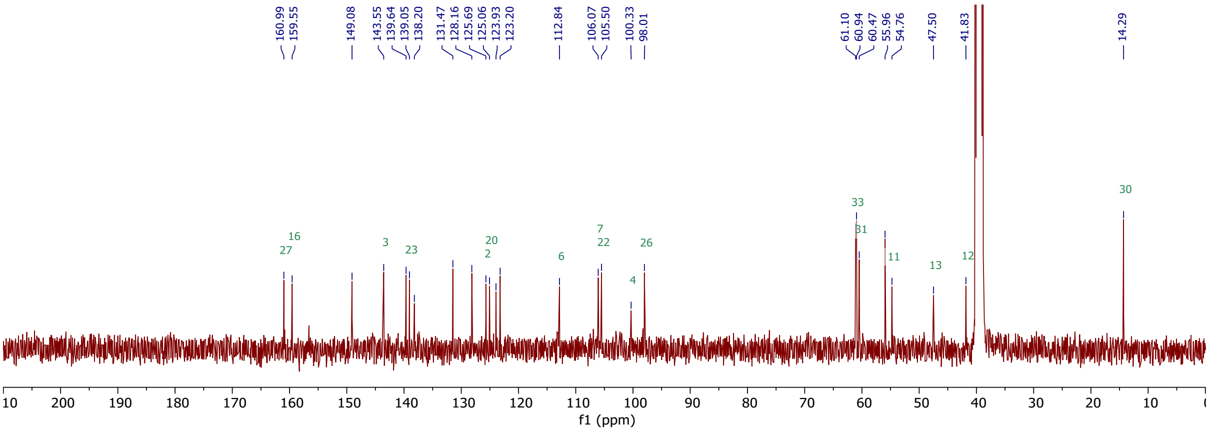

Figure 43. ¹³C-NMR of **11**.

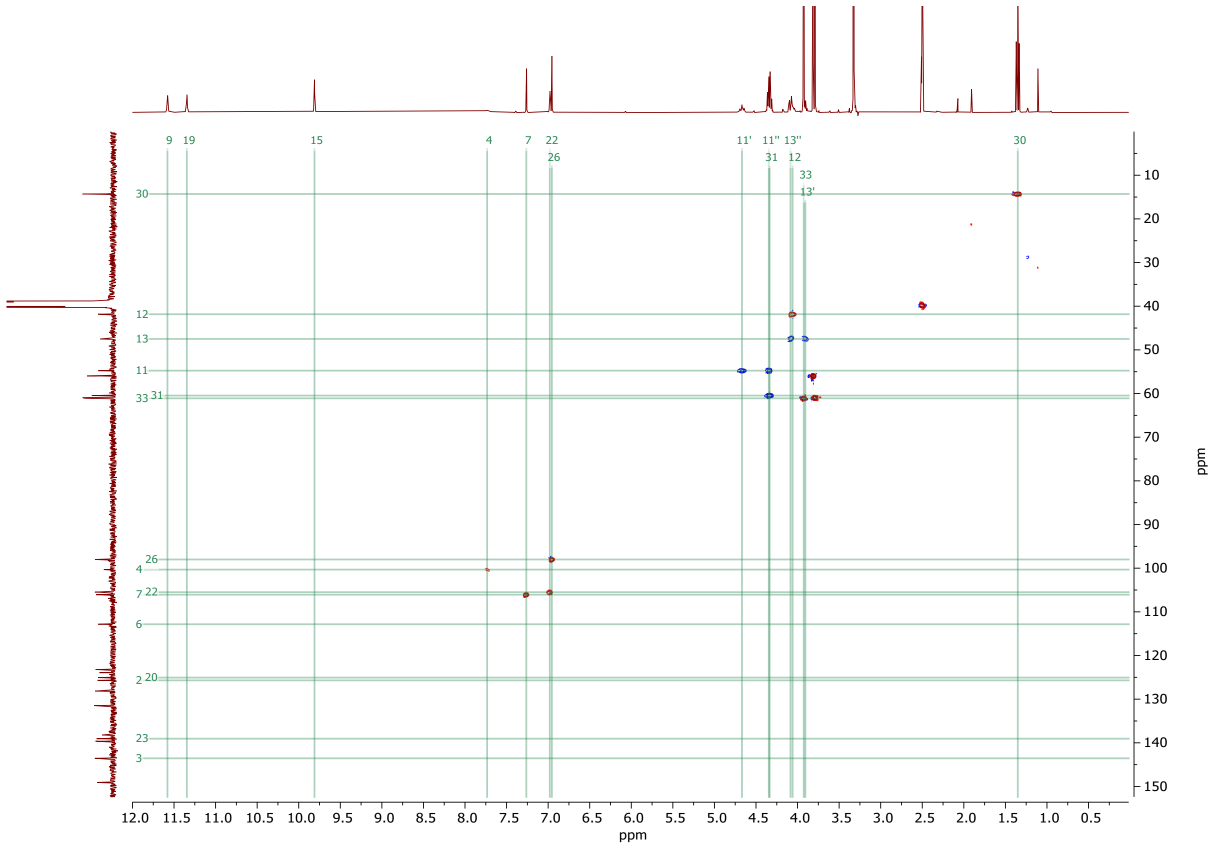

*Figure 44. HSQC of compound **11**.*

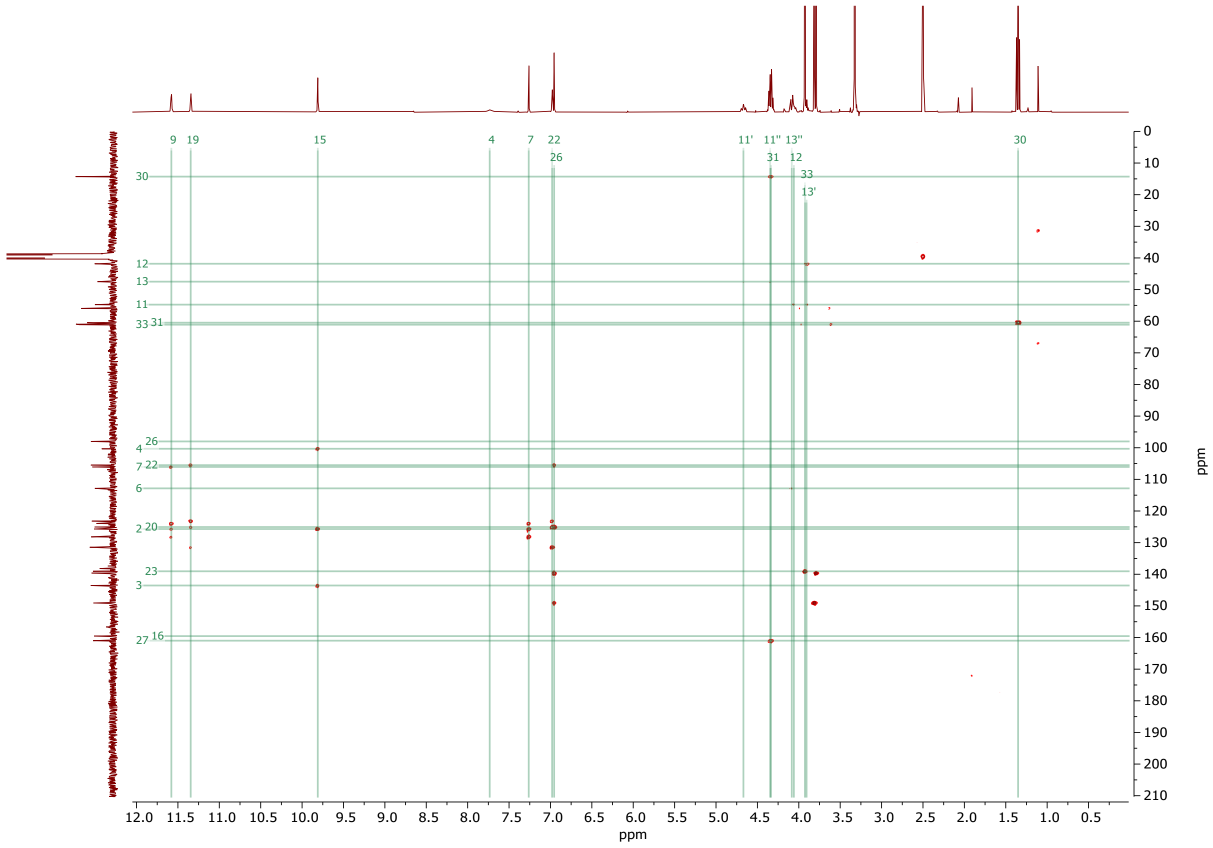

*Figure 45. HMBC of compound **11**.*

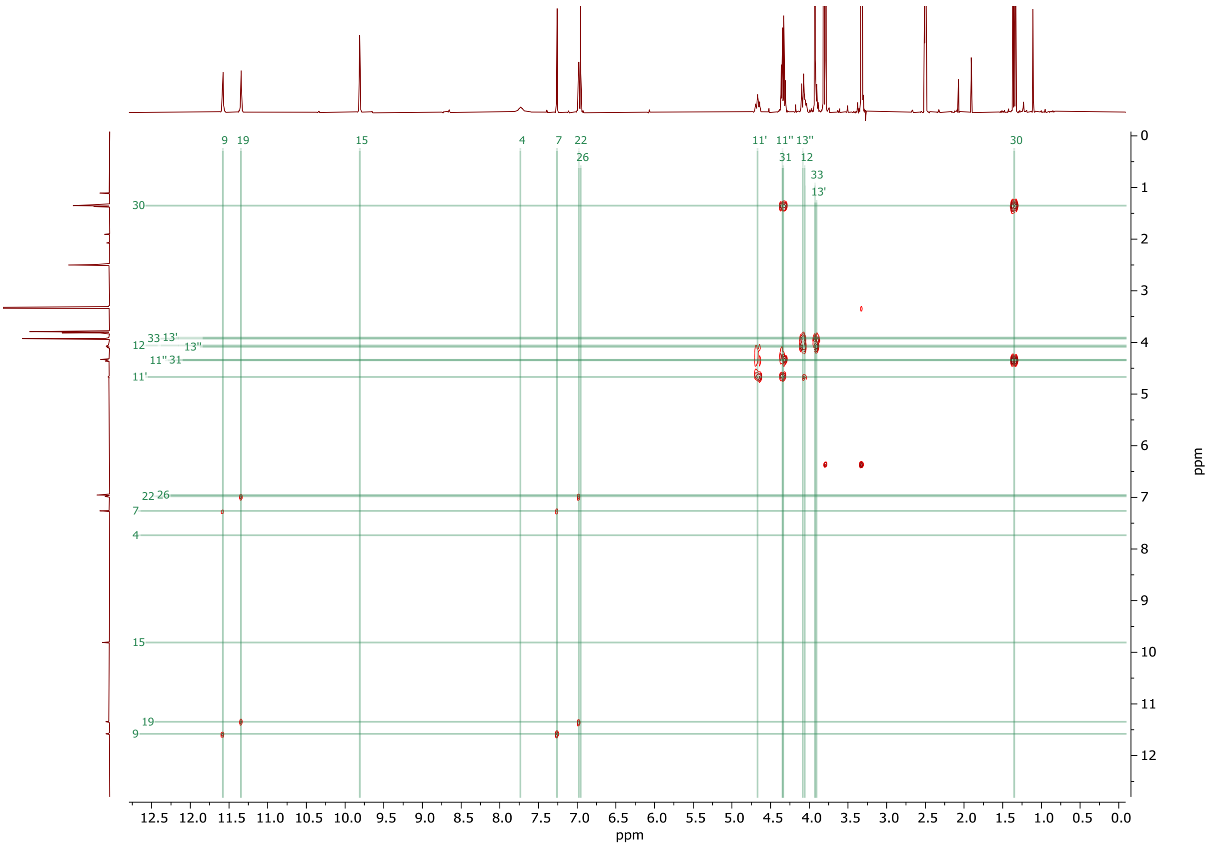

*Figure 46. COSY of compound **11**.*

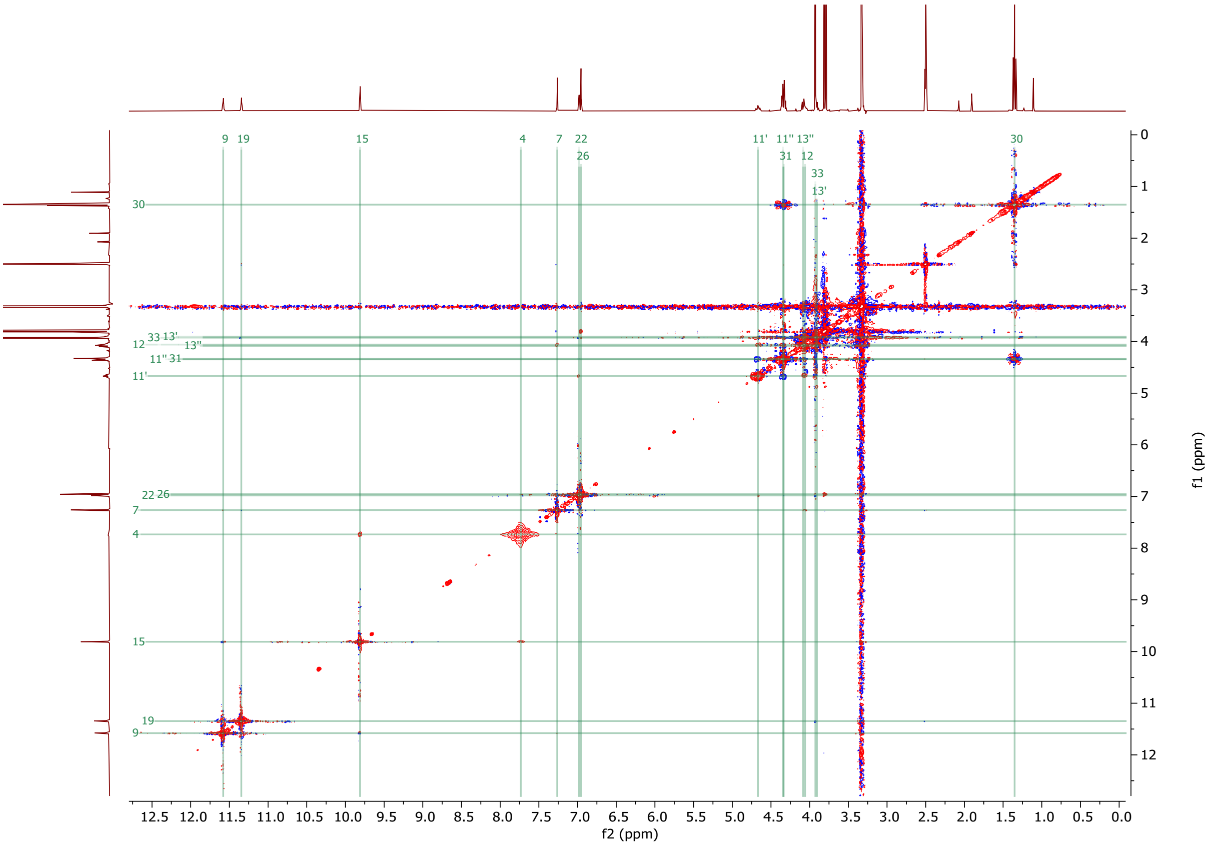

*Figure 47. NOESY of compound **11**.*

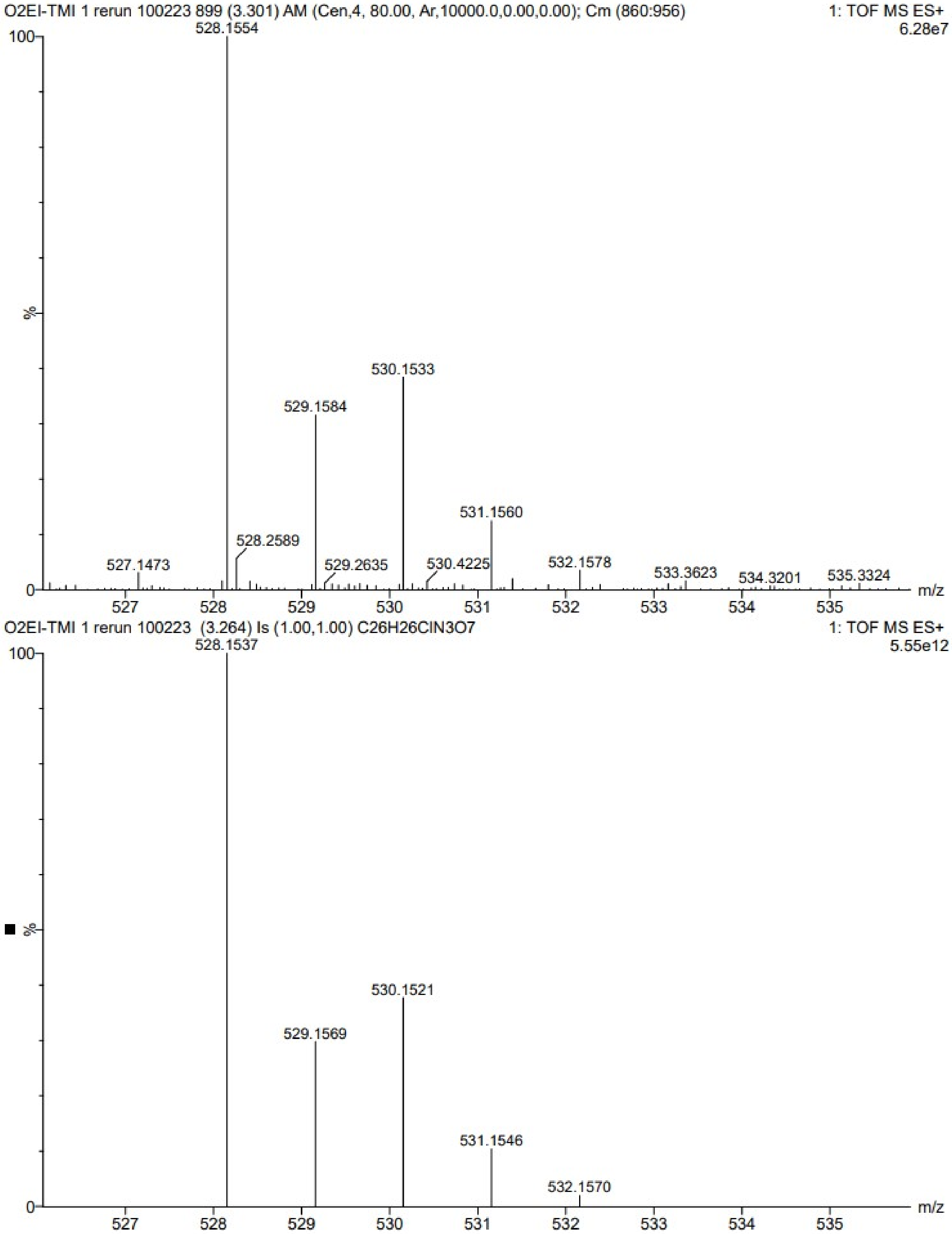

Figure 48. HRMS of compound ***11****.*

Proliferation assay

HL-60 cells were cultured in RPMI media. Cells were seeded at 2x10^5 cells/mL in 96 well plates (100 µL per well). The plates were incubated for 24 hours, before treating with compound (1 µL in DMSO). Doxorubicin (1 µL, 10 µM) was used as a positive control for cell death. The cells were incubated for 72 hours, before treatment with 3-(4,5-dimethylthiaz14ol-2-yl)-5-(3-carboxymethoxyphenyl)-2-(4-sulfophenyl)-2H-tetrazolium (MTS, 10 µL) for 3 hours. The absorption at 492 nm was measured on a BMG Labtech PolarStar Optima plate reader.

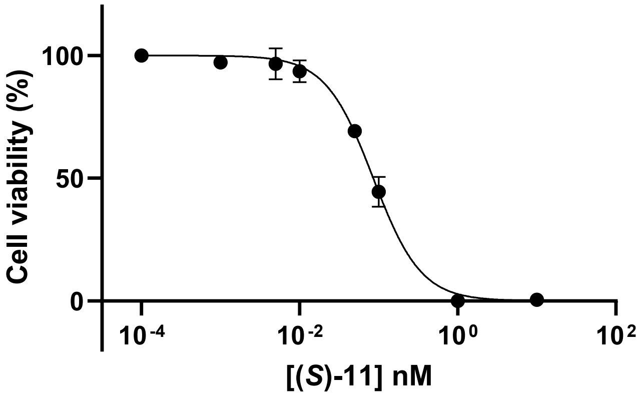

IC$_{50}$: 0.08547 nM [0.07800-0.09434]

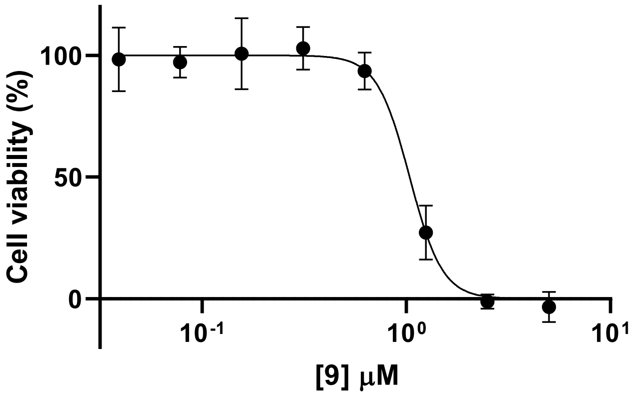

IC$_{50}$: 0.7636 µM [0.6654-0.8924]

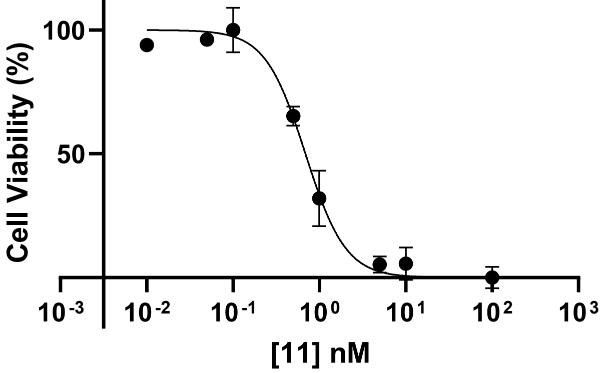

IC$_{50}$: 0.690 nM [0.607, 0.786]

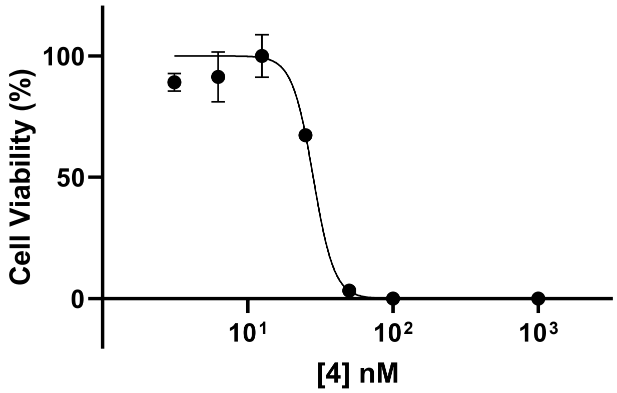

IC_{50}: 26.88 nM [12.83-53.06]
